# Insights into the Biological Activity and Bio‐Interaction Properties of Nanoscale Imine‐Based 2D and 3D Covalent Organic Frameworks

**DOI:** 10.1002/advs.202407391

**Published:** 2024-10-10

**Authors:** Hao Ye, Carlos Franco, Mostafa A. Aboouf, Markus Thiersch, Semih Sevim, Joaquin Llacer‐Wintle, Andrea Veciana, Gemma Llauradó‐Capdevila, Kaiyuan Wang, Xiang‐Zhong Chen, Qiao Tang, Roc Matheu, Pedro D. Wendel‐Garcia, Pedro A. Sánchez‐Murcia, Bradley J. Nelson, Cong Luo, Josep Puigmartí‐Luis, Salvador Pané

**Affiliations:** ^1^ Multi‐Scale Robotics Lab (MSRL) Institute of Robotics & Intelligent Systems (IRIS) ETH Zurich Zurich 8092 Switzerland; ^2^ Institute of Veterinary Physiology Vetsuisse Faculty University of Zurich Winterthurerstrasse 260 Zurich 8057 Switzerland; ^3^ Department of Biochemistry Faculty of Pharmacy Ain Shams University Cairo 11566 Egypt; ^4^ Departament de Ciència dels Materials i Química Física Institut de Química Teòrica i Computacional University of Barcelona Barcelona 08028 Spain; ^5^ Department of Pharmaceutics Wuya College of Innovation Shenyang Pharmaceutical University 103 Wenhua Road Shenyang Liaoning 110016 P. R. China; ^6^ State Key Laboratory of Photovoltaic Science and Technology Shanghai Frontiers Science Research Base of Intelligent Optoelectronics and Perception Institute of Optoelectronics International Institute of Intelligent Nanorobots and Nanosystems Fudan University Songhu Road 2005 Shanghai 200438 China; ^7^ Yiwu Research Intitute of Fudan University Yiwu 322000 China; ^8^ Departament de Química Inorgànica i Orgànica Institut de Química Teòrica i Computacional Barcelona 08028 Spain; ^9^ Institute of Intensive Care Medicine University Hospital Zurich Rämistrasse 100 Zurich 8091 Switzerland; ^10^ Laboratory of Computer‐Aided Molecular Design Division of Medicinal Chemistry Otto‐Loewi Research Center Medical University of Graz Neue Stiftingstalstraße 6/III Graz A‐8010 Austria; ^11^ Institució Catalana de Recerca i Estudis Avançats (ICREA) Pg. Lluís Companys 23 Barcelona 08010 Spain

**Keywords:** cancer therapeutics, cytotoxicity, nanomedicine, nanoscale covalent organic frameworks (nCOFs)

## Abstract

Covalent Organic Frameworks (COFs) emerged as versatile materials with promising potential in  biomedicine. Their customizable functionalities and tunable pore structures make them valuable for various biomedical applications such as biosensing, bioimaging, antimicrobial activity, and targeted drug delivery. Despite efforts made to create nanoscale COFs (nCOFs) to enhance their interaction with biological systems, a comprehensive understanding of their inherent biological activities remains a significant challenge. In this study, a thorough investigation is conducted into the biocompatibility and anti‐neoplastic properties of two distinct imine‐based nCOFs. The approach involved an in‐depth analysis of these nCOFs through in vitro experiments with various cell types and in vivo assessments using murine models. These findings revealed significant cytotoxic effects on tumor cells. Moreover, the activation of multiple cellular death pathways, including apoptosis, necroptosis, and ferroptosis is determined, supported by evidence at the molecular level. In vivo evaluations exhibited marked inhibition of tumor growth, associated with the elevated spontaneous accumulation of nCOFs in tumor tissues and the modulation of cell death‐related protein expression. The research contributes to developing a roadmap for the characterization of the intricate interactions between nCOFs and biological systems and opens new avenues for exploiting their therapeutic potential in advanced biomedical applications.

## Graphical abstract

In this study, the biocompatibility and anti‐neoplastic properties of two imine‐based nanoscale covalent organic frameworks (nCOFs) through in vitro and in vivo experiments are assessed. Results show significant tumor cell cytotoxicity and activation of cellular death pathways. This research contributes to understand the complex interactions between nCOFs and biological systems, opening the door for their therapeutic use in advanced biomedical applications.

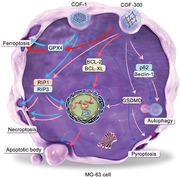

## 1 Introduction

Covalent organic frameworks (COFs) are a unique class of porous crystalline materials characterized by the covalent bonding of organic monomers, resulting in the formation of 2D or 3D reticular structures.^[^ ^1^ ^]^ Owing to their intrinsic porosity and organic composition, these structures offer tremendous potential for biomedical applications.^[^ ^2^, ^3^, ^4^ ^]^ COFs can effectively accommodate various functional chemical cargoes, including diagnostic molecules, contrast agents, and therapeutic compounds, making them promising candidates for improved diagnostics, imaging techniques, and targeted therapies.^[^ ^5^, ^6^, ^7^, ^8^, ^9^, ^10^, ^11^, ^12^ ^]^

Despite significant advancements in the past decade, there are several challenges that need to be addressed for the translation of COFs into clinical applications. A key challenge involves achieving the optimal dimensions.^[^ ^13^ ^]^ Ideally, sizes of therapeutic COFs should be larger than 10 nm to prevent their rapid clearance through the kidneys, while still being smaller than 200 nm in order to evade splenic filtration.^[^ ^14^ ^]^ Additionally, COFs should exhibit sufficient loading capacity to effectively accommodate therapeutic or diagnostic cargoes.^[^ ^11^ ^]^ In this regard, 3D COFs offer distinct advantages over their 2D counterparts. They possess higher surface areas, uniform channel interpenetration, and an increased number of active sites.^[^ ^15^, ^16^ ^]^ However, the synthesis, stability, crystallization, and processability of 3D COFs present significant challenges, resulting in limited availability of these materials to date, with fewer than a hundred reported structures.^[^ ^17^ ^]^ Furthermore, many synthetic procedures fail to produce COFs with optimal sizes, thereby challenging their dispersibility in biological formulations.^[^ ^18^, ^19^, ^20^, ^21^, ^22^ ^]^ Additionally, these procedures often require harsh environments and solvents, hindering their widespread application in biomedicine.^[^ ^23^ ^]^ Another critical issue is the limited scope of cell lines and in vivo studies conducted in COFs research. Therefore, as other therapeutic nanoparticles have been subjected to rigorous evaluation,^[^ ^24^, ^25^ ^]^ COFs similarly require a systematic approach for biocompatibility assessments. This strategy should span across exposure routes, intricate in vitro cytotoxicity tests, and expansive in vivo trials with both healthy and diseased hosts. Addressing these multifaceted challenges is essential for unlocking the full potential of COFs in biomedicine and facilitating their transition into practical healthcare solutions.

Here, we seek to understand the cellular uptake, the cell cytotoxicity, and the in vivo biodistribution of 2D and 3D imine‐based nanoscale COFs (nCOFs). Recently, we reported the synthesis of nano‐sized 2D COF‐1^[^ ^18^ ^]^ and 3D COF‐300^[^ ^26^ ^]^ using a one‐pot method under mild conditions. Specifically, the method involved conducting the process at room temperature, utilizing non‐toxic organic solvents, and capitalizing on micelles as nanosized reaction compartments. Both COFs have a size of ≈35 nm, which is optimal for cellular uptake.^[^ ^27^, ^28^, ^29^ ^]^ Our research reveals that when tested against several normal and tumor cell lines, nCOFs exhibit significant cytotoxicity especially against osteosarcoma MG63 tumor cells and RAW 264.7 macrophages, along with an increased cellular uptake. Several forms of cell death, including ferroptosis, autophagy, necroptosis, and apoptosis were found to be involved. In our study using a healthy mouse model, we observed mild cytotoxic effects associated with nCOFs characterized by slight liver stress and a modest decrease in body weight. Additionally, in MG‐63 tumor‐bearing nude mice model, rapid incorporation of nCOFs into solid tumors was observed, which resulted in a tumor‐inhibiting effect. Our research underscores the significance of extensively assessing the cytotoxicity, cell uptake, and in vivo biodistribution of COFs. Our findings contribute to an in vivo decision‐making framework for COF design and therapeutic applications, ultimately guiding the development of effective COF nanotherapies.

## 2 Results and Discussion

### 2.1 nCOFs Synthesis and Characterization

**Figure 1:**
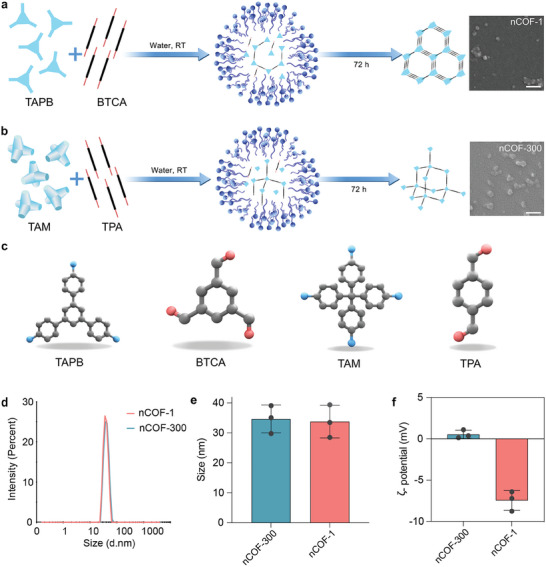
Characteristics of nanoscale covalent organic frameworks (nCOFs). a) Schematic of the preparation process and scanning electron microscope images of nanoscale nCOF‐1 and b) nCOF‐300 (scale bar = 100 nm). Synthesis of nano‐sized 2D nCOF‐1 and 3D nCOF‐300 was achieved via a one‐pot, room‐temperature method using micelles, involving reactions of Benzol‐1,3,5‐tricarbaldehyd (BTCA) with 1,3,5‐Tris(4‐aminophenyl)benzene (TAPB) (nCOF‐1) and tetrakis(4‐aminophenyl)methane (TAM) with terephthaldialdehyde (TPA) (nCOF‐300), respectively. c) The ball‐and‐stick models of TAPB, BTCA, TAM, and TPA. In these models, carbon atoms are depicted as grey spheres, oxygen as red, and nitrogen as blue. d) and e) Size distribution and f) Zeta potential of the prepared nCOF‐1 and nCOF‐300 (*n* = 3, data are presented as mean ± s.e.m.). For Figure 1d, experiments were repeated three times; a representative image is shown.

In this study, we specifically focus on imine‐based COFs, which compared to other kind of COFs such as boronated‐based, present the ability to maintain their structural integrity at standard physiological environments.^[^ ^18^, ^30^ ^]^ Particularly, we capitalized on our one‐pot synthesis to produce COF nanoparticles in an aqueous medium and at room temperature.^[^ ^18^, ^26^ ^]^ This technique involves the confined solubilization of COF's building blocks within the hydrophobic cavities of catanionic micelles. By employing a micellar mixture, we successfully achieve the formation of monodisperse nCOF particles with control over the size, the shape, and the dimensionality. In this particular study, we employed two distinct architectures (**Figure** **1**a–c): a 2D COF, namely COF‐1, and a 3D COF, that is, COF‐300. We produced nanoparticles of COF‐1 (nCOF‐1) by reacting benzene‐1,3,5‐tricarbaldehyd (BTCA) with 1,3,5‐tris(4‐aminophenyl)benzene (TAPB), while nanoparticles of COF‐300 (nCOF‐300) were synthesized through the reaction of tetrahedral tetrakis(4‐aminophenyl)methane (TAM) with linear terephthaldialdehyde (TPA). Further details regarding the materials and methods are described in the supporting information (SI). Note that under these synthetic conditions, nCOFs exhibited a narrow size distribution, as shown in Figure 1d,e. The average hydrodynamic diameter of nCOF‐1 was determined to be 33.8 nm (polydispersity index (PDI): 0.14), whereas nCOF‐300 displayed an average diameter of 34.7 nm (PDI: 0.17), indicating a high degree of uniformity in particle size. Zeta (ζ) potential measurements performed under physiological conditions using PBS (pH 7.4, at room temperature) showed near‐neutral values for both types of nanoparticles. Specifically, the nCOF‐300 displayed a slightly positive value of +0.5 mV, while the nCOF‐1 exhibited a ζ potential of −7.5 mV. This slightly negative values of ζ potential are probably due to the existence of free primary amines^[^ ^31^, ^32^ ^]^ (as detected by infrared spectroscopy, Figure S1, Supporting Information), which are protonated at pH 7.4 (acid dissociation constant (pKa) of amine is in the range of 9–11), which causes the electrostatic attraction of anions of the phosphate‐buffered saline (PBS), that is, phosphate anions (Figure 1f).

These particles were intentionally synthesized to be smaller than 100 nm, as previous research has revealed that nanoparticles with dimensions exhibit enhanced cellular interaction.^[^ ^27^, ^28^, ^29^ ^]^ Note that particles with dimensions below these sizes and comprising negative or neutral surface charges display usually an enhanced circulatory half‐life.^[^ ^33^ ^]^

### 2.2 Cellular Uptake of nCOFs

We evaluated the cellular uptake of nCOFs with various cell lines. To monitor the uptake, we covalently labeled the unreacted amino groups in the nCOFs with the Alexa Fluor 488‐N‐hydroxysuccinimide (NHS) ester. The NHS ester functionality of the dye readily reacts with the NH_2_ groups on COF‐1 and COF‐300, facilitating a robust and stable labeling mechanism and avoiding a burst release of the dye. This labeling technique allowed us to visually track the location of nCOFs within cells using confocal fluorescence microscopy, providing detailed insights into the cellular uptake process (further details in the Materials and Methods Section, Supporting Information).

Based on this labeling strategy, we examined the nCOFs (nCOF‐1 and nCOF‐300) interaction with a variety of cell lines, including mouse macrophages (RAW 264.7), mouse embryo fibroblasts (NIH/3T3), human osteosarcoma cells (MG‐63), human embryonic kidney cells (HEK‐293), and mouse breast cancer cells (4T1). We selected these cell lines because they represent several tissues: macrophages are the most abundant immune cells, fibroblasts are the most important cell type in connective tissue, and kidney cells are key for understanding renal clearance processes. In addition, we selected cancer cells as models, with 4T1 cells representing epithelial mammary carcinoma cells (breast cancer), and osteosarcoma cells because they are derived from mesenchymal cells, which are involved in bone tumorigenesis (osteosarcoma).

**Figure 2:**
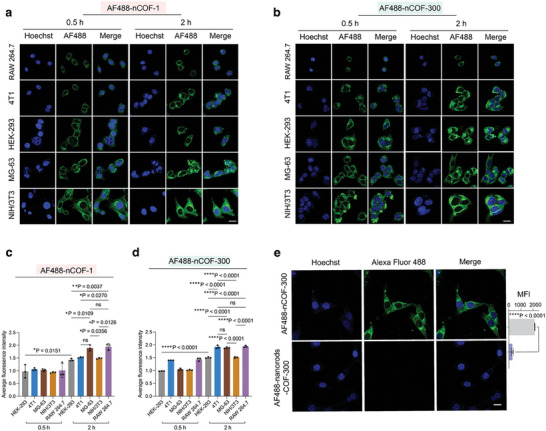
Cellular uptake of nCOFs. a) and b) Confocal laser scanning microscopy (CLSM) images for RAW 264.7, 4T1, HEK‐293, MG‐63, or NIH/3T3 cells after incubating with AF488‐COF‐1 or AF488‐COF‐300 for 0.5 and 2 h, respectively (*n* = 3, Scale bar = 10 µm); c) Fluorescence intensity per unit volume of RAW 264.7, 4T1, HEK‐293, MG‐63, or NIH/3T3 cells by flow cytometry analysis after incubating AF488 (Alexa fluor 488)‐COF‐1 or d) AF488‐COF‐300 (*n* = 3, data are presented as mean ± s.e.m.). e) CLSM imaging and flow cytometry quantify results for MG‐63 cells after incubating with nanoscale or microscale AF488‐labeled‐COF‐300 for 2 h (*n* = 3). Scale bar = 10 µm. Statistical significance was calculated via one‐way ANOVA with a Tukey post–hoc test (Figure 2c,d) or a two‐tailed student's *t*‐test (Figure 2e). ^*^ *p* < 0.05, ^**^ *p* < 0.01, ^***^ *p* < 0.001, ^****^ *p *< 0.0001 versus control.

Our results indicated a time‐dependent cellular uptake of nCOFs. Within 0.5 h of exposure, we observed a noticeable increase in fluorescence signal intensity, suggesting a high particle uptake (**Figure** **2**a–d). Among all the cell types, the highest cellular uptake of nCOF‐1 and nCOF‐300 was observed in NIH/3T3 cells, followed by MG‐63, HEK‐293, 4T1, and RAW 264.7 cells (Figure S2, Supporting Information). Notably, NIH/3T3 cells are 33.7–105.2% larger than the other cells (Figure S3, Supporting Information), potentially accounting for their higher nCOFs uptake. Upon normalizing the fluorescence signal intensity to cell size, osteosarcoma MG‐63 cells and RAW 264.7 macrophages demonstrated the highest relative uptake of nCOFs (Figure 2c,d). Notably, the stability of the labeling strategy employed was confirmed through covalently labeled nCOFs with the two different fluorescent dyes AF488 and AF647 (Figure S4, Supporting Information). The Pearson's correlation coefficient of the dual‐labeled sample is 93.7%, indicating a high degree of colocalization and suggesting that both dyes remain stably associated with the nCOF particles without inducing dye leakage.

Next, we examined the influence of the shape and size of nCOFs on cellular uptake. To this end, we capitalized on the micellar approach, which allows us to tailor the morphology of the COF architectures. Consequently, we synthesized nanorods based on COF‐300, which exhibited a rod‐like morphology, with lengths approximately ranging from 1–2 µm and a width of ≈100 nm (Figure S5, Supporting Information). Importantly, these nanorods retained the identical chemical composition as that of COF‐300. To further investigate the impact of these variations in the cellular uptake, we focused our investigations on MG‐63 osteosarcoma cells due to their pronounced uptake characteristics in our prior observations. Hence, we exposed these cells to both AF488‐labeled nCOF‐300 and COF‐300 nanorods for 2 h (Figure 2e). Notably, the dimensions of the COFs significantly impacted their absorption by cells. Our results demonstrated that MG‐63 cells treated with AF488‐nanorods‐COF‐300 for 2 h showed almost no detectable intracellular fluorescence, indicating minimal cellular uptake of these particles. In stark contrast, when cells were treated with nanoparticulated AF488‐nCOF‐300, a seven‐fold increase in fluorescence signal was recorded (Figure 2e). This difference indicated a preferential and more efficient uptake of smaller, nanoparticulated COFs by the cells, thereby emphasizing the impact of particle size on the cellular uptake efficiency.

### 2.3 Biological Activity and Biocompatibility of nCOFs

After evaluating the nCOFs uptake, we assessed the biocompatibility of nCOF‐1 and nCOF‐300. We exposed NIH/3T3, 4T1, MG‐63, HEK‐293, and RAW 264.7 cells to these nCOFs for 24 and 72 h, analyzing the cell viability by means of MTT (3‐(4, 5‐dimethylthiazolyl‐2)‐2, 5‐diphenyltetrazolium bromide) assay.

**Figure 3:**
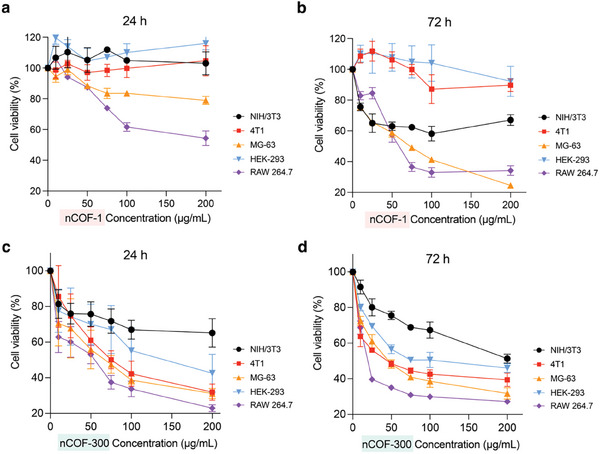
Biocompatibility of nCOFs. a) Inhibitory effects of nCOF‐1 on the proliferation of RAW 264.7, 4T1, HEK‐293, MG‐63, or NIH/3T3 cells for 24 h and b) 72 h incubation as evaluated by MTT assay (*n* = 6, data are presented as mean ± s.e.m.). c) Inhibitory effects of nCOF‐300 on the proliferation of RAW 264.7, 4T1, HEK‐293, MG‐63, or NIH/3T3 cells for 24 h and d) 72 h incubation as evaluated by MTT assay (*n* = 6, data are presented as mean ± s.e.m.).

For nCOF‐1, our data revealed a cell line‐dependent impact on viability. Specifically, MG‐63 and RAW 264.7 cells showed a reduction in viability by 17% and 39%, respectively, when subjected to concentrations exceeding 100 µg mL^−1^ (**Figure** **3**a). However, NIH/3T3, 4T1, and HEK‐293 cells displayed significant viability even at nCOF‐1 concentrations as high as 200 µg mL^−1^ (Figure 3a). Upon prolonged exposure (72 h) to nCOF‐1, we noticed a reduction in the viability of NIH/3T3, MG‐63, and RAW 264.7 cells by 35%, 35%, and 16% respectively at a concentration of 25 µg mL^−1^. Further increasing the nCOF‐1 concentration to 200 µg mL^−1^ triggered a marked decrease in MG‐63 and RAW 264.7 cell viability, dropping by 76% and 66%, respectively (Figure 3b).

In contrast to nCOF‐1, nCOF‐300 exposure resulted in considerable damage to all tested cell lines. After 24 h of incubation with nCOF‐300 at a concentration of 75 µg mL^−1^, viability was significantly reduced across all cell lines: 29% (NIH/3T3), 50% (4T1), 53% (MG‐63), 33% (HEK‐293), and 63% (RAW 264.7) (Figure 3c). Further increasing the nCOF‐300 concentration to 200 µg mL^−1^ resulted in an even more pronounced decrease in viability across all cell lines. These trends persisted following 72 h of nCOF‐300 exposure (Figure 3d).

Considering the significant cytotoxicity of nCOFs to cancerous cells (i.e., MG‐63), we broaden our study to additional cancer cell lines, namely, human A549 and mouse LLC1 lung cancer cells. Similar to our previous findings, these cell lines also decreased cell viability upon exposure to both nCOFs. The consistency of these observations across diverse cancer cell lines highlights the potential cytotoxic effects of nCOFs at higher concentrations (Figure S6, Supporting Information).

### 2.4 nCOFs Activate Multiple Cell Death Pathways

Based on our observations of varied cellular uptake and differing biocompatibility across diverse cell lines, we further investigated the specific cell death mechanisms triggered by nCOFs. Specifically, we examined how nCOFs initiated different cell death pathways, such as apoptosis, necroptosis, autophagy, pyroptosis, and ferroptosis.

Consequently, we exposed cells to specific inhibitors for each death pathway – Z‐VAD‐FMK for apoptosis, Nec‐1 (Necrostatin‐1) for necroptosis, 3‐MA (3‐Methyladenine) for autophagy, Ferrostatin‐1 for ferroptosis, and Disulfiram for pyroptosis – before treating them with different nCOFs for 72 h and measuring their viability.

**Figure 4:**
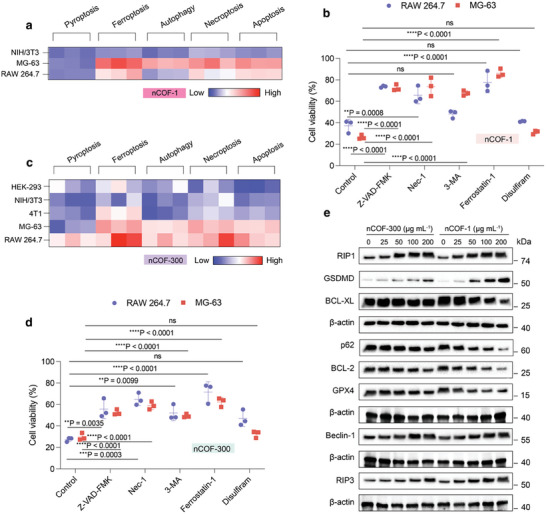
nCOFs induce cell death pathway activation. a) Heatmap of the participation rates of the five cell death modes induced by nCOF‐1 (*n* = 3). b) RAW 264.7 (blue) and MG‐63 (red) cells were pre‐treated with 50 µm Z‐VAD‐FMK, 40 µm Nec‐1, 10 mm 3‐MA, 15 µM Ferrostatin‐1, or 50 µm Disulfiram for 2 h, after 72 h, the cell viability of nCOF‐1 were determined by MTT (*n* = 3, data are presented as mean ± s.e.m.). c) Heatmap of the participation rates of the five cell death modes induced by nCOF‐300 (n = 3). d) RAW 264.7 (blue) and MG‐63 (red) cells were pre‐treated with 50 µm Z‐VAD‐FMK, 40 µm Nec‐1, 10 mm 3‐MA, 15 µm Ferrostatin‐1, or 50 µm Disulfiram for 2 h, after 72 h, the cell viability of nCOF‐300 were determined by MTT (*n* = 3, data are presented as mean ± s.e.m.). e) Expression levels of necroptosis‐related proteins (RIP1 and RIP3), apoptosis‐related proteins (BCL‐2 and BCL‐XL), autophagy‐related proteins (p62 and Beclin‐1), pyroptosis‐related protein (GSDMD), and ferroptosis‐related protein (GPX4) of MG‐63 cells treated with nCOF‐300 and nCOF‐1, respectively. Statistical significance was calculated via one‐way ANOVA with a Tukey post–hoc test (Figure 4b,d). ^*^ *p* < 0.05, ^**^ *p* < 0.01, ^***^ *p* < 0.001, ^****^ *p *< 0.0001 versus control.

For the nCOF‐1, we only focus on NIH/3T3, RAW 264.7, and MG‐63 cell lines, as these cells exhibited relatively high cytotoxicity against this nCOF. Interestingly, NIH/3T3 cells showcased no preferable pathway within all five death modes – pyroptosis, ferroptosis, autophagy, necroptosis, and apoptosis – with modest participation rates across the board (**Figure** **4**a; Figure S7a, Supporting Information). In contrast, MG‐63 and RAW 264.7 cells demonstrated pronounced participation in different cell death pathways upon nCOF‐1 exposure (Figure 4a,b). Particularly, MG‐63 cells activate nearly all cell death modes, with ferroptosis, necroptosis, apoptosis, and autophagy playing the most significant roles. Similarly, RAW 264.7 cells engaged all five cell death modes, with ferroptosis, apoptosis, and necroptosis being the most dominant (Figure 4a,b).

For nCOF‐300, we observed variations in cell death patterns across different cell lines. The involvement in cell death pathways in HEK293, NIH/3T3, and 4T1 cells appeared relatively modest (Figure 4c). Particularly, in HEK293 cells, ferroptosis arise as the dominant mode, while other cell death mechanisms remained negligibly involved (Figure S7b, Supporting Information). For NIH/3T3 cells, mild necroptosis was noticeable (Figure S7c, Supporting Information). In the case of 4T1 cells, ferroptosis was the dominant mechanism, with necroptosis and apoptosis playing a minor role (Figure S7d, Supporting Information). However, analogous to their response to nCOF‐1, both MG‐63 and RAW 264.7 cells displayed significant activation in a variety of cell death pathways when exposed to nCOF‐300. MG‐63 cells displayed again a significant activation across most death modes, with ferroptosis, necroptosis, apoptosis, and autophagy being particularly evident (Figure 4c,d). Likewise, RAW 264.7 cells demonstrated marked activation across all cell death modes, notably with ferroptosis and necroptosis as the predominant pathways.

These nCOF‐1 and nCOF‐300 cell‐death pathways observations indicate distinct cellular responses to nCOFs, especially evident in the MG‐63 and RAW 264.7 cell lines. While these cells demonstrated pronounced activation across multiple cell death pathways, other cell lines showed more modest responses. This disparity emphasizes the inherent sensitivity of MG‐63 and RAW 264.7 cell types to nCOFs and underlines the intricate interplay between nCOF uptake and the subsequent triggering of cell death pathways, ending in varied cytotoxic effects.

Next, we investigated the underlying molecular mechanisms of nCOFs‐induced cell death in MG‐63 cells, considering the potential therapeutic implications for cancer treatment. We assessed the expression levels of seven key proteins associated with different cell death pathways in the presence of nCOFs. Among them, the receptor‐interacting proteins RIP1 (receptor interacting protein kinase‐1) and RIP3 are essential pro‐death signaling molecules in necroptosis. In contrast, BCL‐2 (B‐cell lymphoma 2) and BCL‐XL act as anti‐apoptotic members of the BCL‐2 family. The ubiquitin‐binging scaffold protein p62, a well‐known autophagy receptor, regulates the crosstalk between apoptosis and autophagy through its interaction with Beclin‐1. Gasdermin‐D (GSDMD) mediates cell lysis in pyroptosis, and the enzyme glutathione peroxidase 4 (GPX4) is a central regulator of ferroptosis.

As shown in Figure 4e and Figure S8 (Supporting Information), increased expressions of RIP1 and RIP3 were noted at concentrations as low as 25 µg mL^−1^ for nCOF‐1 and 50 µg mL^−1^ for nCOF‐300, indicating activation of necroptosis. In contrast, the levels of apoptosis inhibitors BCL‐2, BCL‐XL, and autophagy marker p62 began to decline at these concentrations, while the autophagy marker Beclin‐1 demonstrated increased levels. Notably, alterations in the expression of GSDMD, a marker for pyroptosis, and a decrease in GPX4, a ferroptosis marker, were evident at higher concentrations, 50 µg mL^−1^ for nCOF‐1 and 200 µg mL^−1^ for nCOF‐300. Thus, the observed protein expression patterns are consistent with our previous findings, indicating nCOFs' ability to activate several cell death pathways, including ferroptosis, necroptosis, apoptosis, and autophagy. This correlation emphasizes the high sensitivity of MG‐63 cells to both nCOF‐1 and nCOF‐300, highlighting their potential to induce a broad spectrum of cytotoxic responses, as well as suggesting their significant potential as tools in targeted cancer therapy strategies.

### 2.5 In Vivo Pharmacokinetics

Expanding on our in vitro evaluations of cellular uptake and cytotoxicity, we aimed to determine how these outcomes relate to the in vivo performance of nCOFs. To increase the nanoparticles' circulation time and minimize clearance by the reticuloendothelial system, we modified our nCOFs by coating them with non‐toxic and non‐immunogenic polyethylene glycol (PEG) polymer chains. Consequently, the nCOFs were modified using a specific type of PEG, DSPE (1,2‐Distearoyl‐sn‐Glycero‐3‐Phosphoethanolamine)‐PEG2K‐NHS (carboxy N‐Hydroxysuccinimide ester). The NHS group within this PEG variant reacts with the amino groups on the nCOFs surface, creating a covalent bond and, thus, successfully attaching the PEG to the nanoparticle. These modifications led to the creation of PEGylated nCOFs, which were subsequently loaded with the near‐infrared fluorescent dye, 1,1′‐dioctadecyl‐3,3,3′,3′‐tetramethylindotricarbocyanine iodide (DiR), to facilitate in vivo imaging and quantification (further details in the materials and methods section). The resulting nanoparticles, called PEG‐COF‐1@DiR and PEG‐COF‐300@DiR, underwent a shift in their hydrodynamic radius from their original measurements (33.8 and 34.7 nm, respectively) to 47.1 and 47.0 nm (Figure S9, Supporting Information). The zeta potential of PEG‐COF‐1@DiR and PEG‐COF‐300@DiR changed to −2.39 and 6.1 mV, respectively (Figure S10, Supporting Information). Additionally, the thermogravimetric analysis (TGA) curves quantitatively determine the mass percentage of PEG in nCOFs, with PEG‐COF‐1 at 13.1% and PEG‐COF‐300 at 13.2% (Figure S11, Supporting Information). This change, confirmed by DLS and TGA, indicated a successful PEGylation and DiR loading process. Note that the PEGylation of nCOFs did not present significant differences in cellular uptake and cytotoxicity compared to non‐PEGylated nCOFs (Figure S12, Supporting Information), indicating that PEGylation did not significantly affect cell viability. Upon intravenous injection into Sprague‐Dawley rats, DiR exhibited a half‐life of 0.93±0.26 h. As anticipated, the half‐life of DiR increased to 10.97±6.23 h and 7.59±1.84 h when loaded onto PEGylated nCOF‐1 and nCOF‐300, respectively (Figure S13, Supporting Information). Alongside, the maximal DiR plasma concentration increases from 2.47±0.2 ng mL^−1^ to 16.45±2.45 ng mL^−1^ and 12.69±2.13 ng mL^−1^ after the dye was loaded onto PEGylated nCOF‐1 and nCOF‐300, respectively. This data provides evidence that PEGylation extends the circulation time of nCOFs in the body, allowing the PEGylated nCOFs to interact with targeted sites.

### 2.6 nCOFs Performance in Healthy Nude Mice

**Figure 5:**
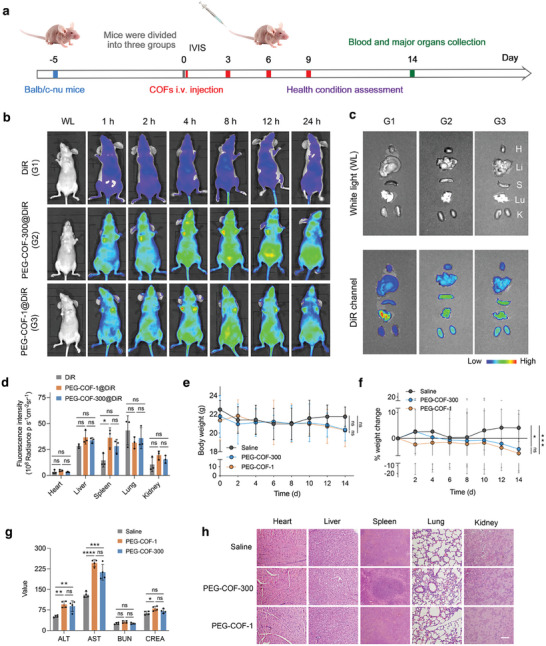
In vivo performance in healthy nude mice model of nCOFs. a) Schematic illustration demonstrating the design of the animal experiments. b) In vivo fluorescence imaging of the healthy mice at 1, 2, 4, 8, 12, and 24 h following injection with PEG‐COF‐300@DiR and PEG‐COF‐1@DiR (*n* = 3). c) Ex vivo fluorescence imaging and d) ROI analysis of major organs obtained 8 h after injection (*n* = 3, data are presented as mean ± s.e.m.). e) Body weight and f) body weight changes (*n* = 5, data are presented as mean ± s.e.m.). g) Hepatorenal function parameters of blood obtained at day 14 (*n* = 5). Data are presented as mean ± s.e.m. h) H&E staining for major organ slices at day 14 (*n* = 3, scale bar = 1 mm). Statistical significance was calculated via one‐way ANOVA with a Tukey post–hoc test (Figure 5d,g) or with Dunnett's multiple comparison test (Figure 5e,f). ^*^ *p* < 0.05, ^**^ *p* < 0.01, ^***^ *p* < 0.001, ^****^ *p* < 0.0001 versus control.

Next, we assessed the biodistribution and potential cytotoxicity of our PEGylated nCOFs within a living system. For this purpose, we selected healthy Balb/c nude mice as our animal model. These mice were systematically administered with intravenous injections of 2 mg kg^−1^ of our PEGylated constructs, specifically DIR loaded nCOFs (PEG‐COF‐1@DiR and PEG‐COF‐300@DiR) were used for in vivo biodistribution imaging. In parallel, non‐DIR loaded nCOFs were applied in vivo toxicity evaluation, conducting injections at regular intervals of 3 days, for 9 days (**Figure** **5**a).

Our observation revealed that the PEGylated nCOFs were quickly distributed across the mouse body within just an hour of injection (Figure 5b). Both PEG‐COF‐1@DiR and PEG‐COF‐300@DiR, along with the DiR dye, remained detectable in the body for up to 24 h. Interestingly, we noticed a shift in the distribution of these substances between 2 to 12 h post‐injection. During this period, PEG‐COF‐1@DiR, and PEG‐COF‐300@DiR were predominantly present in the liver, spleen, and lung, while DiR dye alone was concentrated mainly in the lung and liver (Figure 5c,d).

Continuous monitoring revealed no significant body weight differences between the control and the nCOF‐treated groups until the final injection. However, a decline in body weight was observed in the PEG‐COF‐1 and PEG‐COF‐300‐treated mice, by 8.3% and 5.3%, respectively, by the fifth day post‐final injection, while the control group remained stable (Figure 5e,f). While these reductions are below the critical thresholds typically (20% weight loss) considered in mouse studies,^[^ ^34^, ^35^ ^]^ they indicate an adverse biological reaction to the nCOF particles. Note that, such weight decreases do not serve as definitive indicators of compromised health in the mice but suggest a specific physiological response to the presence of nCOFs.

Following the observed alterations in body weight, we further explored the potential systemic impacts of PEGylated nCOFs, concentrating our examination on liver and kidney functionalities. For this, we measured the concentrations of alanine transaminase (ALT) and aspartate aminotransferase (AST), as elevated concentrations of these enzymes typically indicate liver distress or damage. Concurrently, we assessed blood urea nitrogen (BUN) and serum creatinine (CREA), indicators of kidney function. The results showed a significant elevation in ALT levels for both PEG‐COF‐1 and PEG‐COF‐300‐treated mice, recording values of 95.1 and 88.9 U L^−1^, respectively – values that very close to the upper limit of the standard reference range of 10.1–96.4 U L^−1^.^[^ ^36^ ^]^ In addition, AST concentrations in PEG‐COF‐1‐treated mice reached 246.4 U L^−1^, and PEG‐COF‐300‐treated mice exhibited 212.6 U L^−1^, PEG‐COF‐1 value noticeably exceeding the standard 36.3–235.5 U L^−1^ range,^[^ ^36^ ^]^ which indicates a tendency toward liver stress or low potential damage. However, no significant discrepancies were observed in the levels of BUN and CREA between the nCOFs‐ and saline‐treated mice (Figure 5g), suggesting that the kidney functionality remained unaffected.

Despite the significant alterations in enzyme levels, a meticulous examination of major organs – heart, liver, spleen, lung, and kidney – revealed no observable tissue damage (Figure 5h). These findings suggest that, while PEGylated nCOFs may influence liver function – indicating potential liver stress or injury, as revealed by the elevated ALT and AST levels – major cellular integrity is not compromised by the PEGylated nCOFs. Consequently, their systemic toxicity could be perceived as limited, and their systemic use, give an application‐specific dose‐finding study, appear safe.

### 2.7 nCOFs Performance in Tumor‐Bearing Nude Mice Model

**Figure 6:**
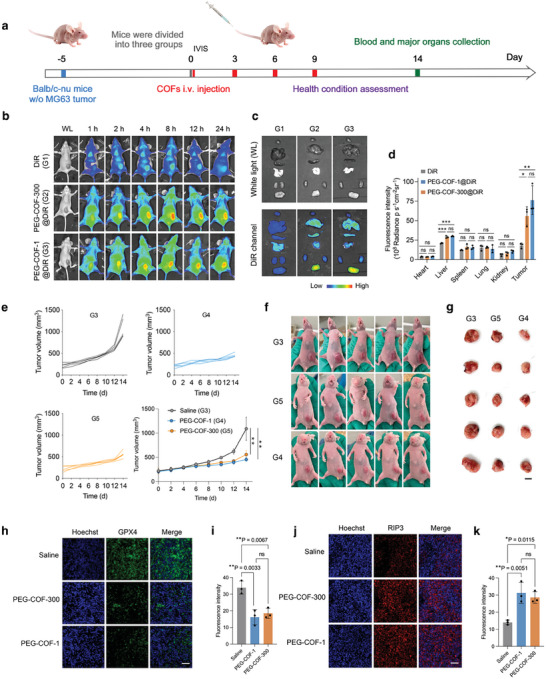
nCOFs performance in MG‐63 tumor‐bearing model. a) Schematic illustration demonstrating the design of the animal experiments. b) In vivo fluorescence imaging of the healthy mice at 1, 2, 4, 8, 12, and 24 h following injection with PEG‐COF‐300@DiR and PEG‐COF‐1@DiR (*n* = 3). c) Ex vivo fluorescence imaging and d) ROI analysis of major organs obtained 8 h after injection (*n* = 3, data are presented as mean ± s.e.m.). e) Tumor growth curves of mice after various treatments (*n* = 5, data are presented as mean ± s.e.m.). f) Photos of MG‐63 tumor‐bearing mice and g) tumors at day 14 (*n* = 5, Scale bar = 7.5 mm).h) Representative immunofluorescence images of GPX4 stained MG‐63 tumor slices and i) fluorescence intensity was quantified using ImageJ software (*n* = 3, Scale bar = 300 µm). Data are presented as mean ± s.e.m. j) Representative immunofluorescence images of RIP3 stained MG‐63 tumor slices and k) fluorescence intensity was quantified using ImageJ software (*n* = 3, Scale bar = 300 µm). Data are presented as mean ± s.e.m. Statistical significance was calculated via one‐way ANOVA with a Tukey post–hoc test (Figure 6d,i,k) or with Dunnett's multiple comparison test (Figure 6e). ^*^ *p* < 0.05, ^**^ *p* < 0.01, ^***^ *p* < 0.001, ^****^ *p* < 0.0001 versus control.

Based on the observed sensitivity of MG‐63 osteosarcoma cells to nCOFs in vitro and the biodistribution patterns identified in healthy mice, we expanded our investigation to assess the biodistribution of these nanoparticles in a tumor‐bearing model. We specifically aimed to discern the targeting tumor capability of PEG‐COF‐1@DiR and PEG‐COF‐300@DiR, ensuring the direct delivery of payloads to tumors under conditions mimicking a clinical setting. To this end, we established a tumor‐bearing mouse model by subcutaneously injecting MG‐63 osteosarcoma cells into nude mice. Upon confirmation of tumor growth, we administered 2 mg kg^−1^ of either PEG‐COF‐1 or PEG‐COF‐300 five days post tumor cell injection, following the same dosing protocol used in earlier stages: injections every three days for nine days. Control mice received either DiR dye alone or a saline solution (**Figure** **6**a).

We observed a significant accumulation of PEG‐COF‐1@DiR and PEG‐COF‐300@DiR in the tumors within an hour of injection. This rapid targeting was in stark contrast to the uniform distribution of DiR dye throughout the bodies of tumor‐free mice (Figure 6b). The accumulation in tumors continued to intensify until the 4‐h mark and was sustained for up to 24 h post‐injection (Figure 6b). Notably, by the 8‐h time point, the accumulation of PEG‐COF‐1@DiR and PEG‐COF‐300@DiR in tumors had significantly surpassed that of the DiR dye in the control mice, being 3.3 and 2.1 times higher, respectively (Figure 6c,d). Furthermore, the concentration of PEG‐COF‐1@DiR and PEG‐COF‐300@DiR in tumors was ≈2.6 and 1.9 times higher, respectively than in the liver, which exhibited the next highest level of nanoparticle concentration (Figure 6c,d).

Our analysis also highlighted a strong correlation between the accumulation of PEG‐COF‐1@DiR and PEG‐COF‐300@DiR in tumors and a marked reduction in tumor growth (Figure 6e–g). Postmortem assessments five days after the final nanoparticle injection showed a significant reduction, over 50%, in tumor weight in the mice treated with PEG‐COF‐1 and PEG‐COF‐300, compared to the saline‐treated controls (Figure S14, Supporting Information). Note that all groups had similar tumor sizes, ≈0.2 cm^3^, before starting the treatment. This observation aligns with the sensitivity and cytotoxicity previously observed in the in vitro studies.

In addition to the observed tumor size reduction, we noticed significant alterations in the expression of key proteins associated with ferroptosis, identified as one of the major cell death pathways in the previous in vitro studies. Specifically, a 2.1‐fold reduction in the expression of GPX4, a critical regulator of ferroptosis, was observed in tumors from mice treated with PEG‐COF‐1, and a 1.8‐fold decrease in those treated with PEG‐COF‐300, compared to saline controls (Figure 5h,i). This decrease in GPX4 was paralleled by an elevation in RIP3 expression, indicative of necroptosis, in tumors from PEG‐COF‐1 and PEG‐COF‐300 treated mice by 2.2‐fold and 2.0‐fold respectively, compared to saline controls (Figure 5j,k). Histopathological analysis of these tumors further highlighted our findings, revealing clear evidence of necrosis. Therefore, these findings, combined with the histological evidence, support the in vitro results and emphasize the capacity of nCOFs to target tumor cells and induce a ferroptosis‐mediated cell death mechanism, leading to the suppression of tumor growth.

Similar with previous observations in tumor‐free mice, we noted that the tumor‐bearing mice that were administered with PEG‐COF‐1 and PEG‐COF‐300 exhibited weight loss after the final nanoparticle injections. This weight reduction was concomitant with a slight rise in plasma AST levels (Figures S15 and S16a,b, Supporting Information), indicative of potential low liver stress. However, an examination of essential organs, including the heart, liver, spleen, lung, and kidney, revealed no apparent signs of tissue damage in these subjects (Figure S16c, Supporting Information). These results are consistent with our preliminary investigations, demonstrating no evident tissue damage and indicating a promising safety profile for our nanoparticle formulations under the tested conditions.

### 2.8 Cellular Uptake Mechanisms

After the observed significant cytotoxic effects of nCOFs against MG‐63 cells in both in vitro and in vivo models, we next analyzed the cellular mechanisms underlying these effects. Given the relevance of the cellular entry mechanisms in mediating such responses, we investigated the endocytosis mechanisms triggered by nCOFs, further complemented by lysosome colocalization studies.

First, we examined the cellular uptake mechanisms of nCOFs by employing inhibitors targeting various endocytic pathways and assessing their specific contributions to nCOF uptake. The study was conducted alongside inhibitors for clathrin‐mediated endocytosis (CME, obstructed with 5 µg mL^−1^ chlorpromazine), caveolae‐mediated endocytosis (CvME, inhibited with 3 µg mL^−1^ indomethacin), and macropinocytosis (MP, impeded with 8 µg mL^−1^ colchicine).

**Figure 7:**
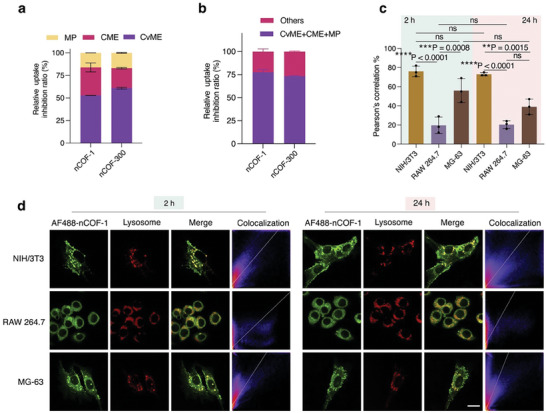
Cellular uptake mechanism of nCOFsc) a) The inhibition of cellular uptake in MG‐63 cells was assessed through flow cytometry data obtained after treatment with numerous endocytosis inhibitors, either independently or b) in conjunction. and subsequent incubation with COF‐1, or COF‐300 (*n* = 3). The inhibitors used pertained to three specific categories: CME, which signifies clathrin‐mediated endocytosis; CvME, representing caveolae‐mediated endocytosis; and MP, for macropinocytosis. The data is illustrated as the mean ± standard error of the mean (s.e.m.). c) The method of Pearson's correlation has been employed for a quantitative evaluation of intracellular colocalization (*n* = 3). Data are presented as mean ± s.e.m. Statistical significance was calculated via one‐way ANOVA with a Tukey post–hoc test (Figure 7c). ^**^ *p* < 0.01, ^***^ *p* < 0.001, ^****^ *p* < 0.0001 versus control. d) Separate channel images exemplifying the co‐localization of AF488‐nCOF‐1 in the lysosome at 2 and 24 h, obtained through Confocal Laser Scanning Microscopy (CLSM) are presented. Yellow fluorescence indicates the overlay areas of the fluorescence‐labeled lysosome (red) and AF488‐nCOF‐1 (green). Scale bar = 10 µm.

Our experimental data revealed that inhibiting CME resulted in a 31.0% and 22.1% reduction in the uptake of nCOF‐1 and nCOF‐300, respectively. Remarkably, the inhibition of CvME induced even more substantial reductions of 52.8% and 60.9% for nCOF‐1 and nCOF‐300, respectively. Conversely, inhibiting MP led to slightly lesser reductions, at 16.2% and 17.0% for nCOF‐1 and nCOF‐300, respectively (**Figure** **7**a). These results underscore that while CME and MP are significant routes for nCOF uptake, CvME emerges as the dominant pathway for the internalization of nCOFs. This is evidenced by the pronounced reductions in nCOF uptake upon inhibition of CvME.

Interestingly, the inhibition of these three pathways resulted in a 70% reduction in the uptake of both nCOF‐1 and nCOF‐300 (Figure 7b), underscoring the predominance of endocytosis as the principal mechanism for nCOF internalization in MG‐63 cells. This finding emphasizes the significance of endocytosis in COFs’ cellular uptake while indicating other, clearly less prominent, uptake mechanisms.

Subsequently, further insights into the uptake mechanism were taken through lysosome colocalization studies, providing valuable information into the complex interactions between cells and nCOFs. The CytoPainter lysosomal staining dye, known for its ability to selectively stain lysosomes within living cells, was employed. This facilitated real‐time observation of the co‐localization of nCOFs with lysosomes, thus elucidating their intracellular trajectories and the involvement of endocytosis‐mediated uptake mechanisms. For this analysis, the response of MG‐63 cells was compared with that of NIH/3T3 and RAW 264.7 cell lines, considering their varied responses noted in prior in vitro studies. Notably, RAW cells manifested significant cytotoxicity against nCOFs, similar to MG‐63, while NIH/3T3 cells, despite exhibiting the most pronounced total nCOF internalization, displayed enhanced biocompatibility.

Afterward, the selected cell lines were incubated with nCOFs for 2 or 24 h, followed by co‐staining with the CytoPainter dye. Notably, NIH/3T3 fibroblasts exhibited marked co‐localization between nCOF‐1 and lysosomes at both 2 and 24‐h intervals (Figure 7c,d), indicating a lysosome endocytosis‐mediated uptake of nCOF‐1 particles. This interpretation was reinforced by an ≈80% correlation between nCOF‐1 and CytoPainter signals during these periods. In contrast, the MG‐63 osteosarcoma cells displayed only partial co‐localization of nCOF‐1 and CytoPainter, as evidenced by a correlation ≈50% (Figure 7c). Moreover, RAW 264.7 macrophages exhibited minimal co‐localization between AF488‐nCOF‐1 and CytoPainter at both 2‐ and 24‐h post‐incubation with nCOF‐1, with a correlation being a mere 20% (Figure 7c).

Having established that nCOFs could be internalized into cell's lysosomes, assessing their intracellular stability is crucial, especially considering the harsh acidic conditions typically encountered within these cellular organelles.^[^ ^37^ ^]^ To assess this, we simulated the lysosomal environment by incubating nCOFs in an acetic acid solution at pH 4.5 and 37 °C for 24 h. This simulation resulted in a partial degradation of nCOFs, evidenced by a decrease in particle diameter from ≈40  to ≈8 nm. This size reduction is indicative of the hydrolysis of the imine (C═N) linkages, which are the essential bonds that constitute the structural integrity of the nCOFs (Figure S17, Supporting Information).

Consequently, this alteration in structural stability and accelerated degradation, induced by lysosome‐mediated internalization of imine nCOFs, hold significant implications for their potential cytotoxicity. The different trends in cell viability align with the insights derived from the studies on endocytosis‐mediated uptake and lysosomal co‐localization. Remarkably, NIH/3T3 cells, characterized by significant nCOF‐lysosome co‐localization, appear to use this association as a defensive mechanism, mitigating the cytotoxic effects induced by nCOFs and thereby showing better biocompatibility even under elevated nCOF concentrations. In stark contrast, RAW 264.7 and MG‐63 cells, despite their high nCOF uptake, exhibited a reduced degree of nCOF‐lysosome co‐localization. This diversity indicates a potential accumulation of undegraded nCOFs within these cells, inducing the activation of various cell death pathways and resulting in increased cytotoxicity.

Accordingly, the intricate interplay between nCOF uptake and lysosomal degradation emerges as a critical factor in the cellular response to nCOF exposure and the resultant cytotoxicity. The findings from this study reveal the complexity of the relationship between nCOF uptake, lysosomal degradation, the activation of cell death pathways, and resultant cytotoxicity, underscoring the multifaceted nature of these cellular interactions.

## 3 Conclusion

In conclusion, this study reveals the multifaceted interactions of nanoparticulated COFs (nCOFs) with biological systems, exposing their selective cytotoxicity, cellular interactions, and promising potential in cancer therapeutics. Our investigation with nCOFs shows the influence of particle size on cellular uptake, the activation of diverse cell death pathways, and the cell‐type specificity of their interactions.

A closer exploration of cellular uptake mechanisms revealed the dominance of endocytosis in nCOF uptake. In addition, the critical interplay between nCOF uptake, lysosomal degradation, and the activation of diverse cell death pathways was demonstrated to be essential in understanding the cellular responses to nCOF exposure.

In vivo evaluations evidenced the rapid distribution and pronounced tumor accumulation of nCOFs associated with marked tumor growth inhibition. Despite some indications of potential liver stress, the absence of evident tissue damage suggests a moderated systemic toxicity profile, reinforcing the potential therapeutic application of these nanoparticles. However, further optimization (i.e., concentration, degradation, surface modification, etc.) should be accurately performed to fully exploit the biological potential of nCOFs.

In summary, our findings significantly advance understanding the intricacies of using nCOFs in biomedical applications. These insights make a notable contribution to the current knowledge in the field and establish a solid basis for further research. Our results deepen the comprehension of the cellular dynamics of nCOF interactions and provide a blueprint for optimizing nanoparticle design, enhancing therapeutic applications, and addressing potential adverse effects.

## Conflict of Interest

The authors declare no conflict of interest.

## Supporting information



Supporting Information

## Data Availability

The data that support the findings of this study are available in the supplementary material of this article.
