# Supplementary material for: Insights into the Biological Activity and Bio‐Interaction Properties of Nanoscale Imine‐Based 2D and 3D Covalent Organic Frameworks
Source: Adv Sci (Weinh). 2024 Oct 10;11(44):2407391. doi: 10.1002/advs.202407391 (PMC11600295; doi:10.1002/advs.202407391)
Supplement: Supplementary file 1 — Supporting Information [file ADVS-11-2407391-s001.docx]

Supporting Information

Insights into the biological activity and bio-interaction properties of nanoscale imine-based 2D and 3D covalent organic frameworks

Hao Ye^1^, Carlos Franco^1*^, Mostafa A. Aboouf^2,3^, Markus Thiersch^2^, Semih Sevim^1^, Joaquin Llacer-Wintle^1^, Andrea Veciana^1^, Gemma Llauradó-Capdevila^4^, Kaiyuan Wang^5^, Xiang-Zhong Chen^6,7^, Qiao Tang^1^, Roc Matheu^8^, Pedro D. Wendel-Garcia^9^, Pedro A. Sánchez-Murcia^10^, Bradley J. Nelson^1^, Cong Luo^5*^, Josep Puigmartí-Luis^4,11*^, Salvador Pané^1*^

*^1^*Multi-Scale Robotics Lab (MSRL), Institute of Robotics & Intelligent Systems (IRIS), ETH Zurich, Zurich 8092, Switzerland.

*^2^*Institute of Veterinary Physiology, Vetsuisse Faculty, University of Zurich, Winterthurerstrasse 260, 8057 Zurich, Switzerland.

^3^Department of Biochemistry, Faculty of Pharmacy, Ain Shams University, 11566 Cairo, Egypt.

*^4^*Departament de Ciència dels Materials i Química Física Institut de Química Teòrica i Computacional, University of Barcelona, Barcelona 08028, Spain

*^5^*Department of Pharmaceutics, Wuya College of Innovation, Shenyang Pharmaceutical University, 103 Wenhua Road, Shenyang Liaoning, 110016, P. R. China

*^6^*Institute of Optoelectronics, Fudan University, Songhu Road 2005, Shanghai, 200438, China

^7^Yiwu Research Intitute of Fudan University, Yiwu China.

*^8^*Departament de Química Inorgànica i Orgànica, Institut de Química Teòrica i Computacional, Barcelona 08028, Spain

^9^Institute of Intensive Care Medicine, University Hospital Zurich, Zurich, Switzerland

^10^Laboratory of Computer-Aided Molecular Design, Division of Medicinal Chemistry, Otto-Loewi Research Center, Medical University of Graz, Neue Stiftingstalstraße 6/III, A-8010, Graz, Austria

^11^Institució Catalana de Recerca i Estudis Avançats (ICREA), Pg. Lluís Companys 23, 08010 Barcelona, Spain

*Correspondence and requests for materials should be addressed to carlos.franco@chem.ethz.ch (C.F.); luocong@syphu.edu.cn (C.L.); josep.puigmarti@ub.edu (J.P.L.); vidalp@ethz.ch (S.P.).

**EXPERIMENTAL SECTION**

**Materials.** Tetrakis(4-aminophenyl)methane (TAM), and 1,3,5-Tris(4-aminophenyl)benzene (TAPB) were sourced from Tokyo Chemical Industry, while Terephthaldialdehyde (TPA), and Benzene-1,3,5-tricarbaldehyd (BTCA) were from Sigma-Aldrich. DiR, 3-(4,5-dimethylthiazol-2-yl)-2,5-diphenyltetrazolium bromide (MTT) were supplied by Sigma-Aldrich. Cell culture reagents were provided by Gibco and Hoechst from Thermo Fisher Scientific. All solvents and reagents utilized were of analytical standard grade unless otherwise stated.

**Synthesis of COF-300.** The fabrication of COF-300 requires initial preparation of solutions A and B. For Solution A, 132.2 mg of TA (CAS: 60532-63-0) is first dissolved in 320 ml of DMSO using ultrasound radiation. This solution is then gradually introduced into a 96 ml solution of CTAB 0.1M while applying sonication. After 10 minutes of sonication at room temperature, a yellow dispersion forms. Following this, an additional 4 ml of a 0.1M SDS solution is incorporated and sonicated for another 2-3 minutes. Lastly, 1 ml of pure acetic acid is added to yield a clear yellow solution. For Solution B, dissolve 94 mg of TPA (CAS: 623-27-8) in 200 ml of DMSO. Gradually add this solution into a 96 ml solution of CTAB 0.1M while using sonication. After 10-20 minutes of sonication at room temperature, a clear dispersion forms. Add 4 ml of a 0.1M SDS solution and sonicate for another 2-3 minutes, resulting in a clear, transparent dispersion.

To synthesize COF-300 nanoparticles, begin by combining solution A and solution B, followed by the addition of 2.5 ml of pure acetic acid. Allow this reaction to proceed for 48 hours within a temperature range of 37-40 ºC. Following this period, integrate 200 ml of EtOH into the reaction mixture and let it interact for about 20-30 minutes. At the conclusion of this timeframe, a yellow dispersion should be evident. Proceed to isolate the nanoparticles using centrifugation and purify them through repeated redispersion in additional EtOH; execute this process approximately 3-4 times or until all the surfactants are eliminated. Finally, redisperse the nanoparticles in water or in a buffer of your choice.

For the fabrication of COF-300 fibers, a similar process is employed. Combine solution A and solution B, then introduce 5 ml of pure acetic acid. Permit the reaction to continue for 48 hours at a temperature between 37-40 ºC. Subsequently, add 200 ml of EtOH to the reaction mixture and allow it to react for about 20-30 minutes. By the end of this period, a yellow dispersion will have formed. The nanoparticles should then be isolated by centrifugation and purified by redispersing them in more EtOH. This step is to be repeated 3-4 times or until all surfactants are removed. The final step involves redispersing the fibers in water or a selected buffer.

**Synthesis of COF-1.** The process for synthesizing COF-1 begins by dissolving 23.3 mg of BTCA in 0.25 mL of DMSO. This resultant solution is gradually introduced into 58 mL of a 0.1 M aqueous CTAB solution under the influence of ultrasound. At first, a white cloudy suspension appears, but it soon re-dissolves. Following this, 1.8 mL of a 0.1 M aqueous SDS solution is added. Meanwhile, a separate mixture is created by dissolving 50.6 mg of TAPB in 0.25 mL of DMSO. Similar to the previous procedure, this solution is slowly added to 58 mL of a 0.1 M aqueous CTAB solution while being subjected to ultrasonication. Initially, a white cloudy suspension forms, but it rapidly re-dissolves. Subsequently, 1.8 mL of a 0.1 M aqueous SDS solution is included. Next, the two aqueous solutions are combined and supplemented with 5.8 mL of acetic acid. The blend is deoxygenated through three vacuum-argon cycles and allowed to react at 30℃ for a period of 72 hours, resulting in a fully transparent orange colloidal solution. To obtain the final product in solid form, the reaction mixture is neutralized with 6.8 mL of concentrated ammonia and 100 mL of ethanol, which leads to the emergence of a yellow solid. The dispersion is then centrifuged for 3 minutes at 1500 rcf, with the supernatant being removed. The remaining solid is reintroduced into 50 mL of ethanol and stirred for half an hour before undergoing another round of centrifugation. This cleaning procedure is performed eight times in total, yielding a highly concentrated ethanol dispersion. Finally, the sample is activated through a process known as critical point drying.

**Characterization of nCOFs.** Images of COF-1 and COF-300 were obtained using a Zeiss Ultra 55 field-emission scanning electron microscope (FE-SEM) at an acceleration voltage of 5.0 kV. Prior to imaging, the samples were prepared by applying a Pt/Pd coating via a Quorum Q150T-S sputter coater. For the nCOFs degradation analysis, a buffer solution was prepared with a pH of 4.5 using glacial acetic acid and DI water. A known amount of each nCOF (1 mg) was dispersed in the prepared acetic acid buffer. The suspensions were then incubated at 37°C for 24 hours to simulate the lysosomal environment. Following the incubation period, the size and size distribution of the nCOFs were measured by dynamic light scattering (DLS) using a Malvern Zetasizer. The experiment was performed in triplicate to ensure the reliability and reproducibility of the results.

**Cell culture.** Murine macrophage cells (RAW 264.7), mouse embryo fibroblasts (NIH/3T3) were grown under Dulbecco’s Modified Essential Medium (DMEM, 31966021, Thermo Fisher Scientific, Gibco). Human osteosarcoma cells (MG-63), human embryonic kidney cells (HEK-293), and human non-small cell lung adenocarcinoma cells (A549) were cultured in Minimum Essential Medium (MEM, 41090028, Thermo Fisher Scientific, Gibco). Mouse breast cancer cells (4T1) and mouse Lewis lung carcinoma cells (LLC1) were maintained in RPMI medium 1640 (21875034, Thermo Fisher Scientific, Gibco). All cultured cells were supplied by the American Type Collection (ATCC) and all the medium were enriched with 10% FBS, streptomycin (100 μg/mL), as well as penicillin (100 units/mL). We cultured the cells under 5% CO_2_ at 37 °C.

**Cellular uptake and cytotoxicity assessments.** To explore cellular uptake of the COF nanoparticles, we seeded the RAW 264.7, 4T1, MG-63, HEK-293, and NIH/3T3 cells in 35 mm dishes (Thermo Scientific, 15235672) for 24 h. For fluoresent labelling, 10 mg of nCOFs in 1 mL of 5 mM HEPES buffer (pH 7.5) and sonicating the mixture for 2 minutes. Fluorescent labeling was achieved by dispersing 100 μL of 0.1 mg/mL Alexa Fluor 488-NHS ester from Thermo Fisher Scientific (product number A20000) dissolved in anhydrous DMSO was added, and the solution was stirred for 1 hour at room temperature. The dye-marked nCOFs were then isolated by centrifugation for 15 minutes at 20,000g and room temperature and were subsequently washed four times with 2 mL deionized water to eliminate any unbound dye. Then, dye-labelled COF-300, and COF-1 (50 μg mL^-1^) were incubated with cells for 0.5 h or 2 h, respectively. Cells were then washed thrice in cold PBS, and nuclear was labelled using hoechst 33342 (Invitrogen R37165). Confocal laser scanning microscopy (CLSM, Zeiss LSM 880 airyscan, Germany) was perfomed to observe the cells, and flow cytometry (LSRFortessa, BD Biosciences) was used to quantify the cellular uptake. To gain more insight into the mechanisms underlying cellular uptake, MG-63 cells were first subjected to various endocytosis inhibitors for a duration of 1 h, either independently or in conjunction. Subsequently, these cells were exposed to nCOFs marked with Alexa Fluor 488 and incubated for a period of 2 h. The outcomes pertaining to intracellular fluorescence were subsequently evaluated using the FACSCalibur flow cytometry technique.

For cell viability tests, HEK-293, RAW 264.7, MG-63, A549, and LLC1 cells were seeded at a density of 3×10^4^ cells/mL for a 24 h MTT assay and 1×10^4^ for a 72 h MTT assay whereas NIH/3T3 and 4T1 cells were seeded at a density of 2.5×10^4^ for a 24 h MTT assay and 0.75×10^4^ for a 72 h MTT assay in a 48-well plate. The well plates were incubated for 24 h and then treated with different samples. To explore the cell death mechanisms, cells were pre-incubated with/without 50 μM Z-VAD-FMK, 40 μM Nec-1, 10 mM 3-MA, 15 μM Ferrostatin-1, or 50 μM Disulfiram for 2 h. After that, cells were treated with different samples for 72 h. The MTT assay was employed to determine the cell viability. 20μl of 5mg/ml MTT solution in PBS was added to each well and plates were incubated for 4h in the dark at 37°C, followed by replacing the medium with 200μl DMSO to dissolve formazan crystals. The Varioskan Flash multimode microreader was employed to quantify the absorbance of the samples.

To investigate the co-localization of nCOFs within the lysosomes of NIH/3T3, RAW, and MG-63 cells, we incubated these cells with AF488-COF-1 for either 2 or 24 hours. Lysosomes were distinguished by labeling with Lysosomal Staining Kit-Red-Cytopainter (ab112137), while AF488-COF-1 was identified through a green marker; their overlapping presence manifested as yellow fluorescence. Quantitative analysis of intracellular co-localization was performed using ImageJ software and Pearson's correlation method, based on three independent biological replicates (n=3).

**Western blot analysis.** After being treated with COF-1 and COF-300 (25, 50, 100, 200 μg mL^-1^) for 72 h, the MG-63 cells were harvested and lysed in a lysis buffer (Beyotime) on an ice bath, then centrifuged at 9000 rpm for 8 min. The concentration of the protein were detected via a BCA kit (Dingguo, China). Isolation of total cellular proteins was accomplished with a protein extraction kit (Dingguo, China) for the characterization of membrane protein. Fractionation of the isolated proteins was conducted using an SDS-PAGE method. Afterward, the gel was stained with Coomassie blue. Isolation of proteins for western blot was performed as described previously^1^. After that, the proteins were transfer-embedded onto polyvinylidene fluoride (PVDF) membranes (Bio-Rad). Proteins were blocked for 1 h with 5% skim milk, and then incubation of the membrane with primary antibodies at 4 °C against RIP1 (610459, BD Transduction Laboratories), RIP3 (17563-1-AP, Proteintech), BCL2 (A19693, Abclonal), BCL-XL (A19703, Abclonal), p62 (A19700, Abclonal), Beclin-1 (A17028, Abclonal), GSDMD (A20728, Abclonal), GPX4 (A1933, Abclonal), GAPDH (A19056, Abclonal) performed overnight, respectively. Further, incubation of the membranes with secondary antibody for 1 h at room temperature. Further, cells were incubated with secondary antibody for 1 h. The Chemi-Doc system^+^ Image Lab software (Bio-Rad) was applied for analysis.

**Animal studies.** The mice were maintained at the Animal Center of Shenyang Pharmaceutical University (China). All study procedures, as well as experiments, were carried out in accordance with the following guidelines and approved by the Institutional Animal Ethical Care Committee (IAEC) of the university.

***In vivo* models evaluation.** To establish the tumor-bearing mice model, 1 × 10^7^ MG-63 cells were subcutaneously on the lower right flank of female Balb/c nude mice. nCOFs were first modified with DSPE-PEG2K-NHS (Xi’an ruixi Biological Technology Co., Ltd), and then loaded the DiR for in vivo imaging. To functionalize nCOFs with DSPE-PEG2K-NHS for subsequent DiR loading, the COFs were first washed with 100 mM HEPES buffer at pH 7.4 to deprotonate the amine groups on their surface. Following this preparation, DSPE-PEG2000-NHS, dissolved in DMSO, was reacted with the COFs at a 2:1 molar excess of PEG to surface amine groups. The reaction mixture was incubated overnight with agitation. Post-functionalization, the PEG-nCOFs conjugates were purified by washing three times with water to remove any unbound PEG, using centrifugation at 16,000g for 10 minutes, and then briefly sonicated for uniform dispersion. For DiR loading, the PEGylated COFs were incubated with a DiR solution in DMSO and stirred overnight. The resulting PEG-COF@DiR complexes were isolated by centrifugation at 8,000 rpm for 10 minutes, washed thoroughly with an ethanol:water (3:1) mixture to eliminate non-incorporated dye. When the tumor volume reached 300-350 mm^3^, the mice were intravenously injected with nCOFs (tumor bearing mice model and healthy mice, 2 mg kg^-1^ DiR). Fluorescence imaging was explored at different time points (1 h, 2 h, 4 h, 8 h, 12 h, 24 h) via in vivo imaging system. The mice sacrificed at 8 h, collecting tumors and major organs for further quantitative analysis.

For *in vivo* tumor treatment, MG-63 tumor bearing mice were divided into three groups (n = 5) after the tumor volume reaching 200 mm^3^. From day 0, PEG-COF-300 and PEG-COF-1 (2 mg kg^-1^) were injected to the mice every 3 d for 9 d, respectively (tumor bearing mice model and healthy mice). The tumor volume and mice body weight were measured every 2 d. At day 14, the blood was obtained for hepatic and renal function analysis, and major organs were collected for H&E staining evaluation. For the immunofluorescence analysis, tumors were harvested for the frozen section. Then the slices were incubated overnight with Anti-RIP3 antibody (Abclonal, A5431) or Anti-GPX4 antibody (Abclonal, A1933), respectively. Secondary antibody FITC Goat anti-rabbit IgG (Sigma Aldrich, AP132F) or TRITC Goat anti-rabbit IgG (Abclonal, AS040) were added to the samples and incubated for 1 h, followed by washing and nuclear staining. All slices were imaged by CLSM and quantified by ImageJ.

***In vivo* pharmacokinetics.** 15 Sprague-Dawley rats were divided into three groups randomly and intravenously injected with DiR, PEG-COF-1@DiR, PEG-COF-300@DiR, respectively (DiR, 1 mg kg^-1^). At the predetermined time points (0.25, 0.5, 1, 3, 6, 8, 12, and 24 h), blood sample was obtained and DiR were extracted by 4 volumes of methanol, and the concentration of DiR was measured by Varioskan Flash multimode microreader (Ex: 748 nm, Em: 780 nm).

**Statistical analysis.** All results are indicated as mean ± standard error of the mean (s.e.m.). One-way ANOVA (Tukey post-hoc tests) were applied for multiple comparisons and the student’s t-test was employed for pairwise comparisons. The survival rate was assessed using the log-rank test. All statistical analyses were conducted using the Prism software package (PRISM 8.0.2; GraphPad Software, 2019). P < 0.05 indicated statistical significance.


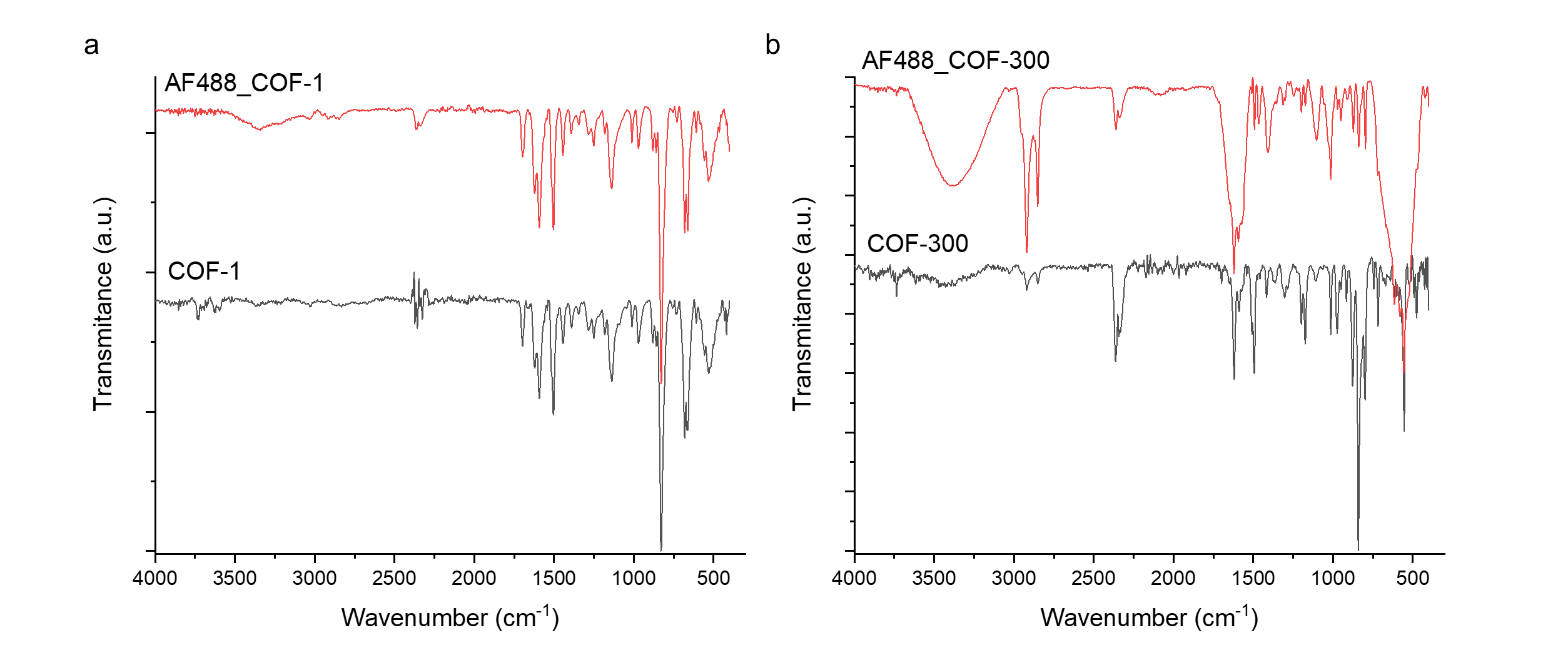


**Figure S1.** FTIR comparison between AF488-labeled and non-labeled nanoCOFs. a) nCOF-1; b) nCOF-300.


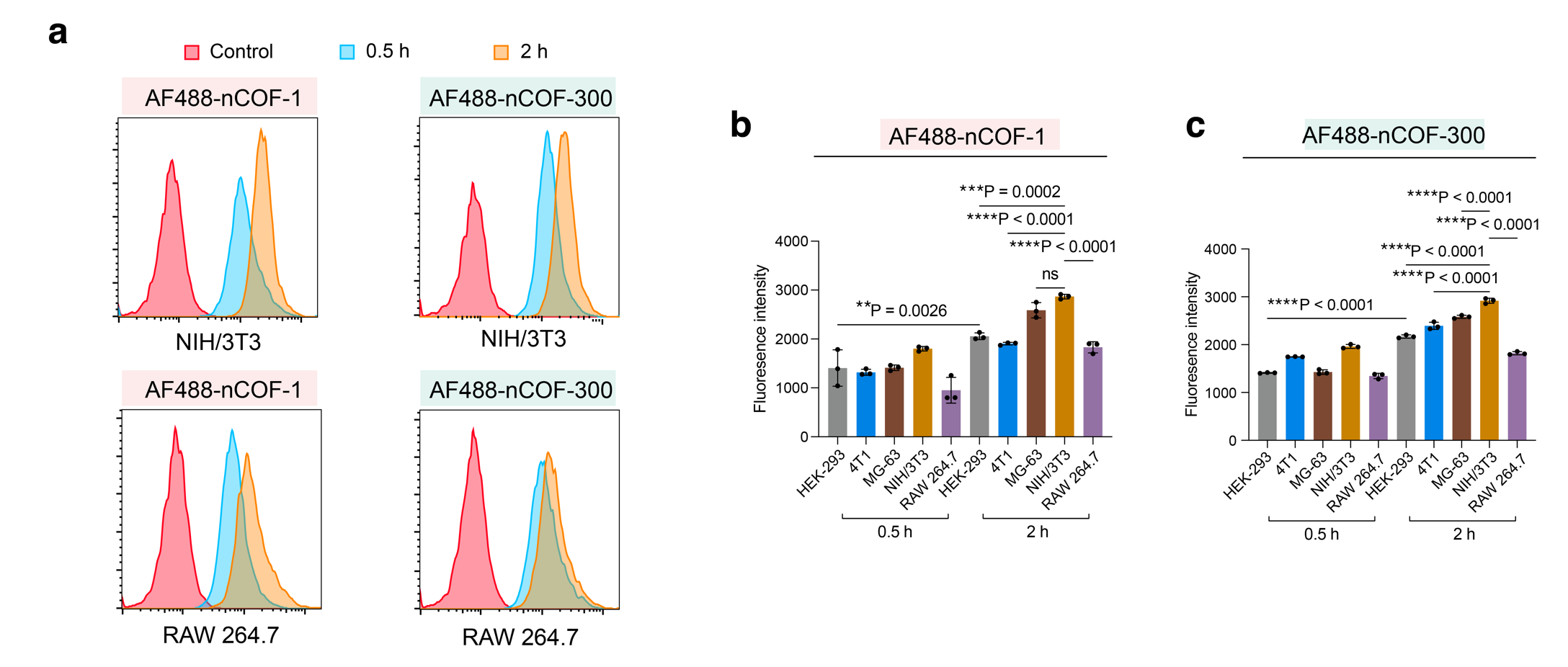


**Figure S2.** Cellular uptake of nCOFs. a) Representative flow cytometry assessment for NIH/3T3 and RAW 264.7 cells after 0.5 h and 2 h of incubation with AF488-nCOFs (n = 3). b) Flow cytometry quantify results for RAW 264.7, 4T1, HEK-293, MG-63, or NIH/3T3 cells after incubating AF488-COF-1 or c) AF488-COF-300 (n = 3, data are presented as mean ± s.e.m.). Statistical significance was calculated via one-way ANOVA with a Tukey post-hoc test (Fig. S2b,c).* p < 0.05, ** p < 0.01, ***p< 0.001, **** p< 0.0001 versus control.


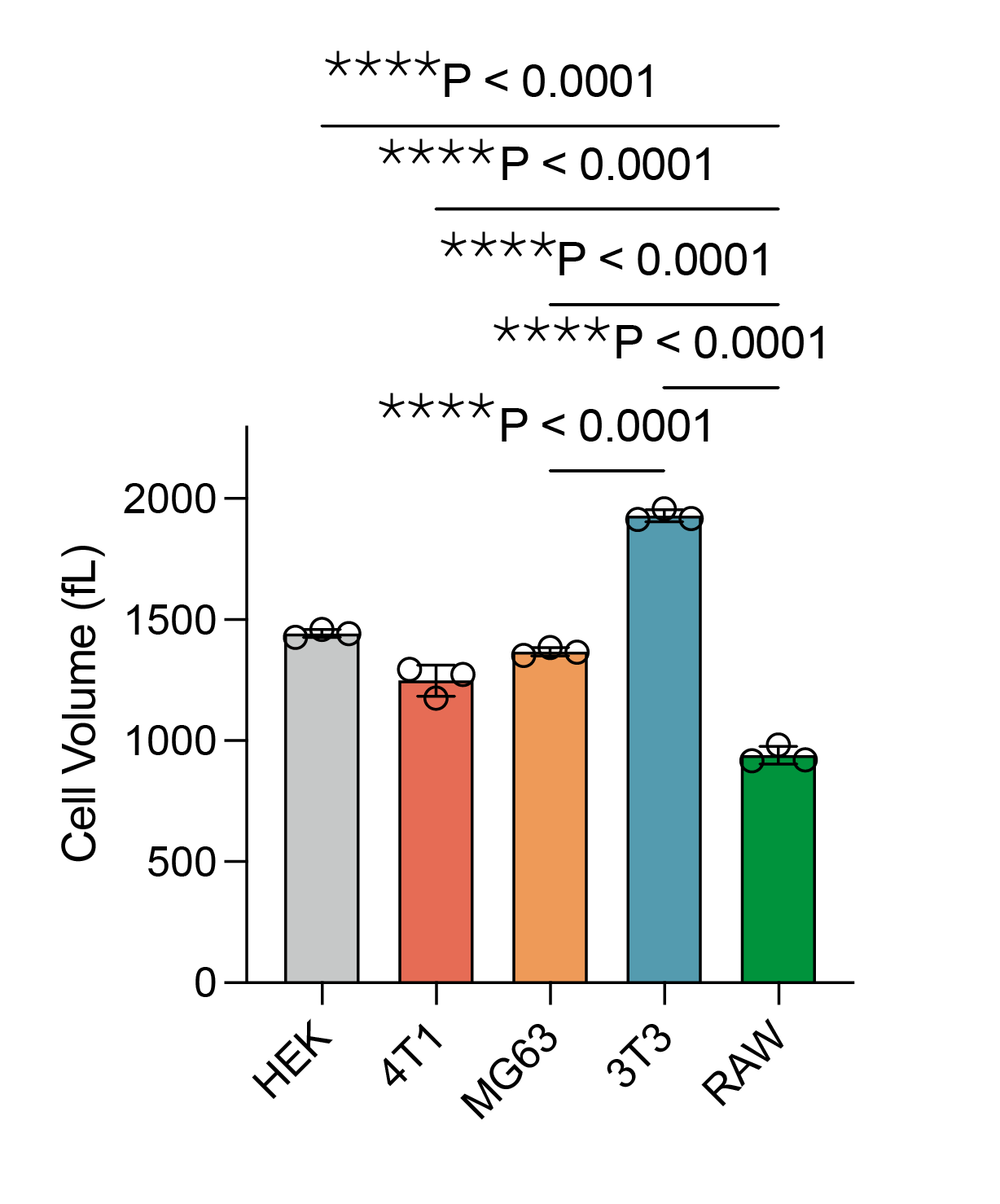


**Figure S3.** Cell volume of HEK293, 4T1, MG-63, NIH/3T3, and RAW 264.7 cells by coulter counter analysis (n = 3, data are presented as mean ± s.e.m.). Statistical significance was calculated via one-way ANOVA with a Tukey post-hoc test. **** p< 0.0001 versus control.


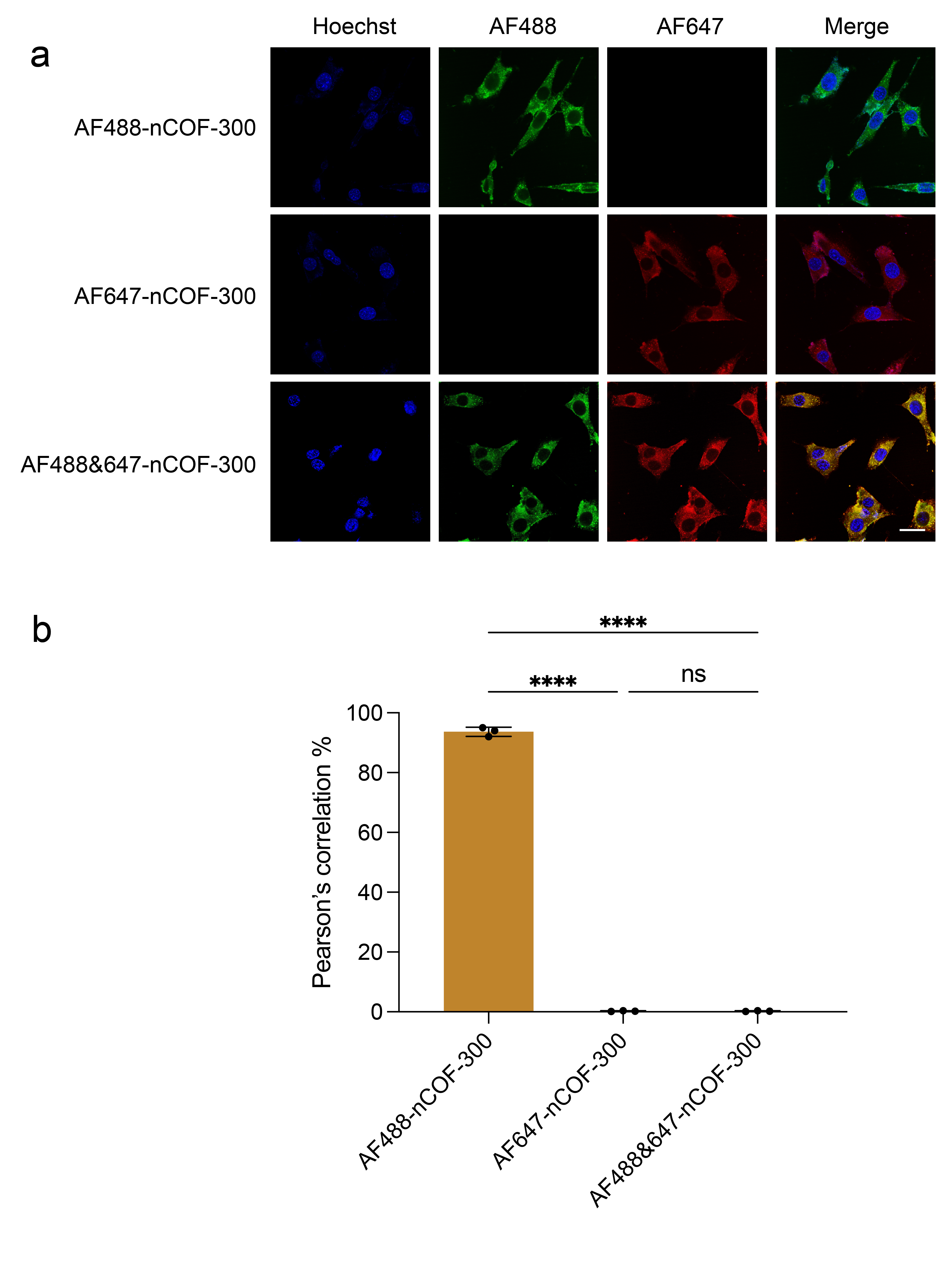


**Figure S4.** Cellular uptake of dye-labeled nCOFs. a) Separate channel images exemplifying the co-localization of AF488 and AF647 of AF488&647-nCOF-300 at 2 hours incubation with NIH/3T3 cells, obtained through Confocal Laser Scanning Microscopy (CLSM) are presented. Yellow fluorescence indicates the overlay areas of AF647 (red) and AF488 (green). Scale bar = 10 μm. b) The method of Pearson's correlation has been employed for a quantitative evaluation of intracellular colocalization (n = 3). Data are presented as mean ± s.e.m. Statistical significance was calculated via one-way ANOVA with a Tukey post-hoc test. **** p< 0.0001 versus control.


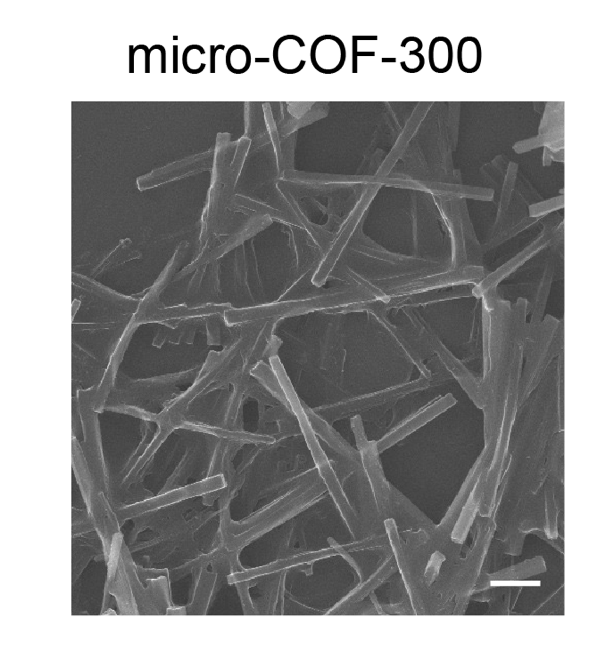


**Figure S5.** SEM images of microscale COF-300 fiber (Scale bar = 1 μm).


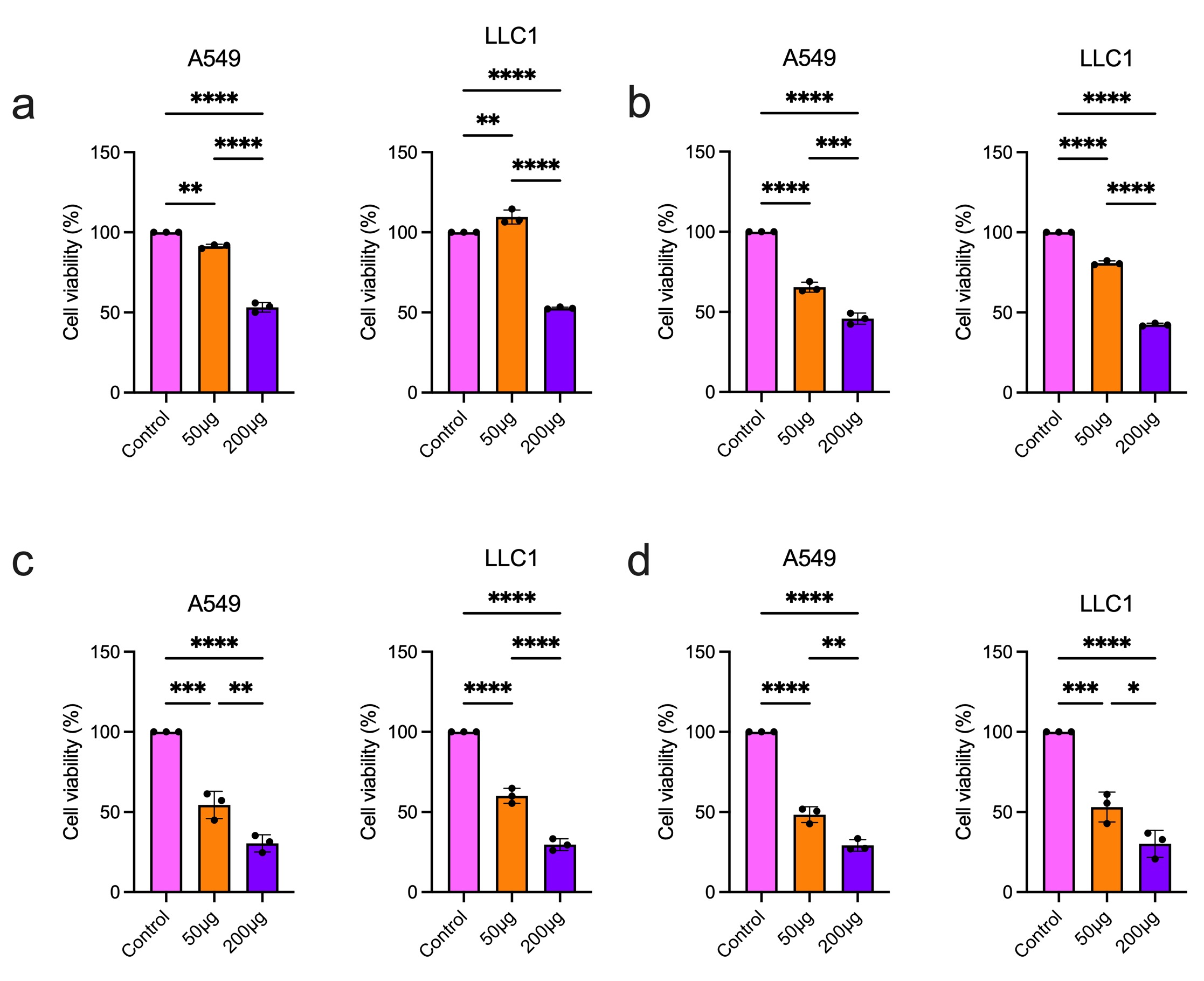


**Figure S6.** Inhibitory effects of COF-1 on the proliferation of human A549 and mouse LLC1 lung cancer cells for a) 24 h and b) 72 h incubation as evaluated by MTT assay (n = 3, data are presented as mean ± S.D.). Inhibitory effects of COF-300 on the proliferation of human A549 and mouse LLC1 lung cancer cells for c) 24 h and d) 72 h incubation as evaluated by MTT assay (n = 3, data are presented as mean ± S.D.).Statistical significance was calculated via one-way ANOVA with a Tukey post-hoc test. ** p < 0.01, *** p < 0.001**** p< 0.0001 versus control.


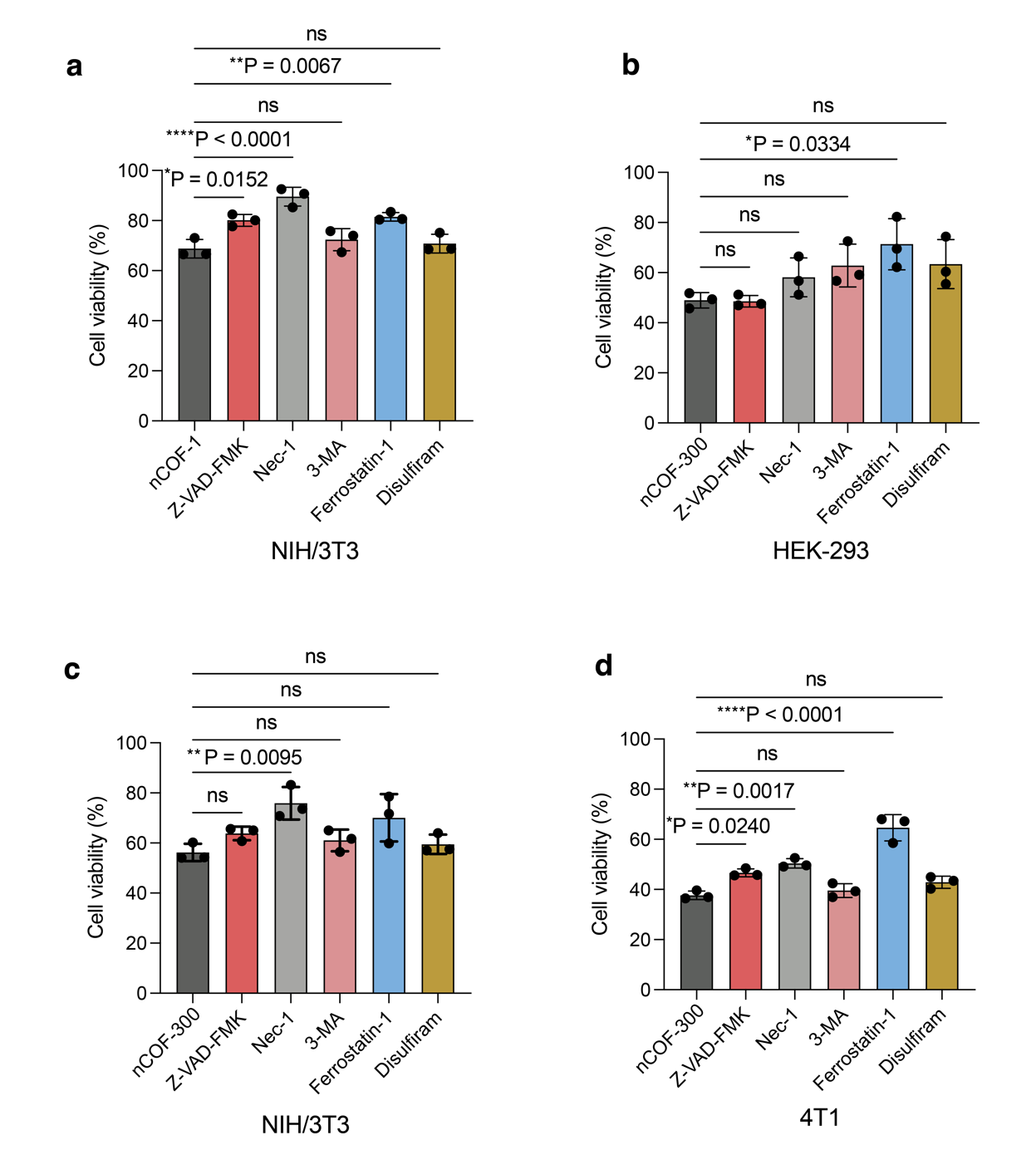


**Figure S7.** a)-d) NIH/3T3, HEK293, 4T1 cells were pretreated with 50 μM Z-VAD-FMK, 40 μM Nec-1, 10 mM 3-MA, 15 μM Ferrostatin-1, or 50 μM Disulfiram for 2 h, after 72 h, the cell viability of COF-300 or COF-1 were determined by MTT (n = 3, data are presented as mean ± s.e.m.). Statistical significance was calculated via one-way ANOVA with a Tukey post-hoc test. * p < 0.05, ** p < 0.01, **** p< 0.0001 versus control.


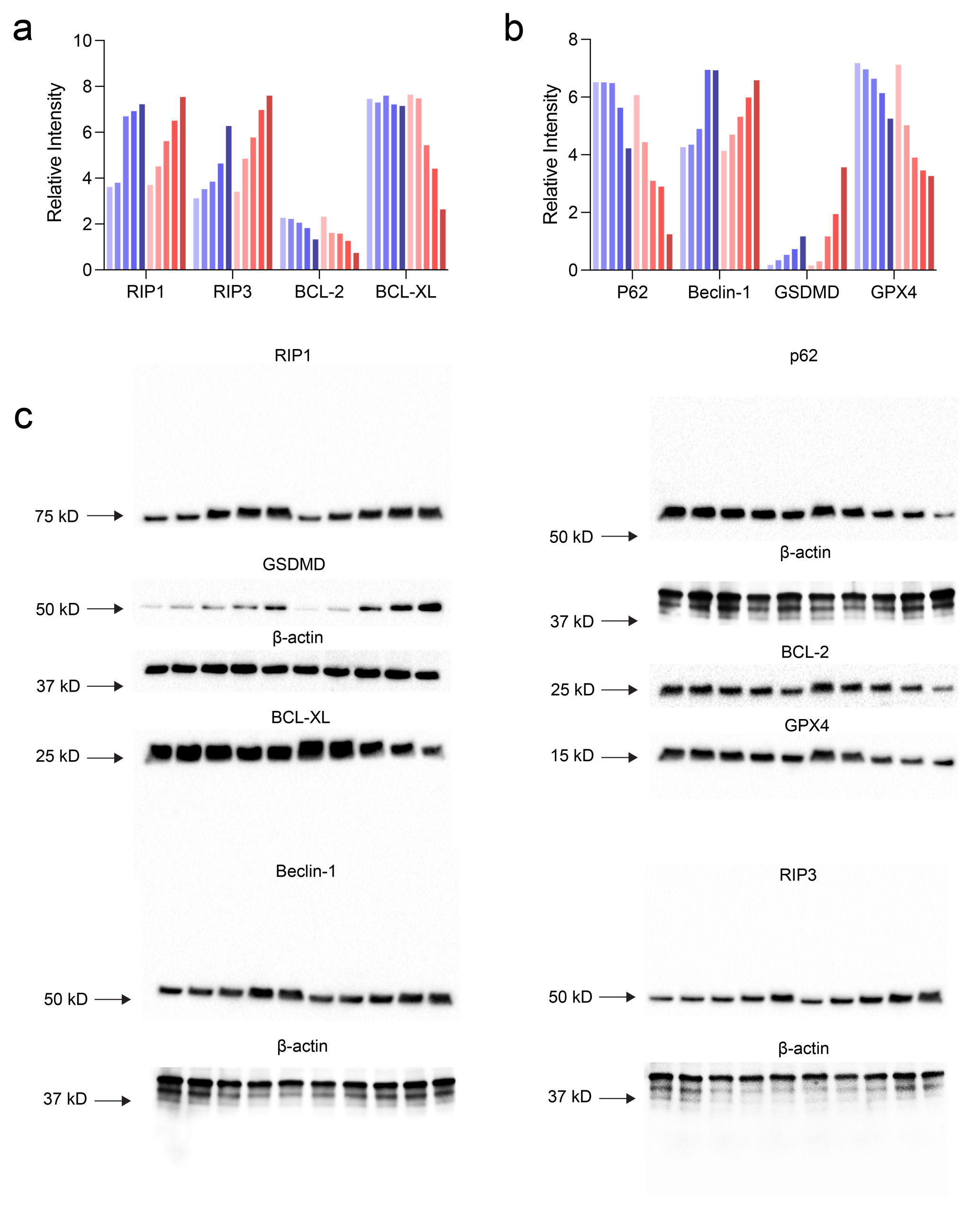


**Figure S8.** a) The histograms of the relative intensity of cell death pathway related proteins RIP1, RIP3, BCL-2, BCL-XL and b) P62, Beclin-1, GSDMD, and GPX4. c) Uncropped western blots for Figure 4e.


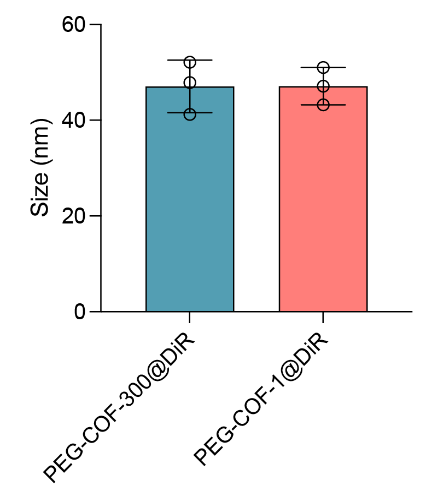


**Figure S9.** Size of PEG-COF-1@DiR, and PEG-COF-300@DiR (n = 3, data are presented as mean ± s.e.m.).


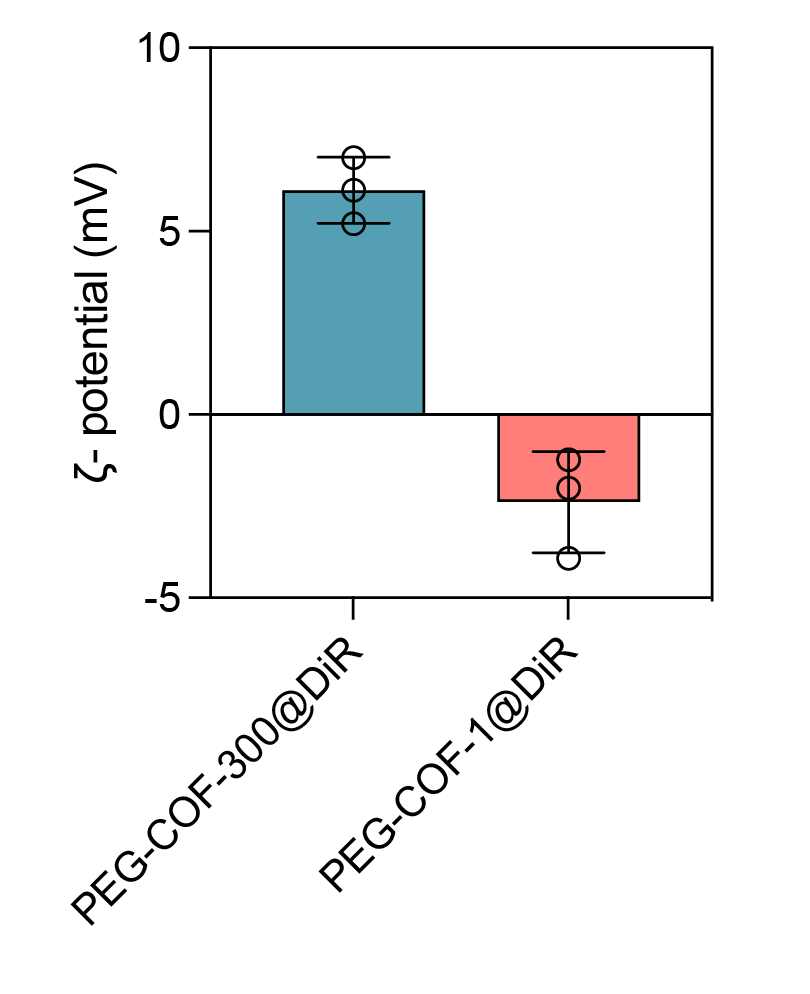


**Figure S10.** Zeta potential of PEG-COF-1@DiR, and PEG-COF-300@DiR (n = 3, data are presented as mean ± s.e.m.).


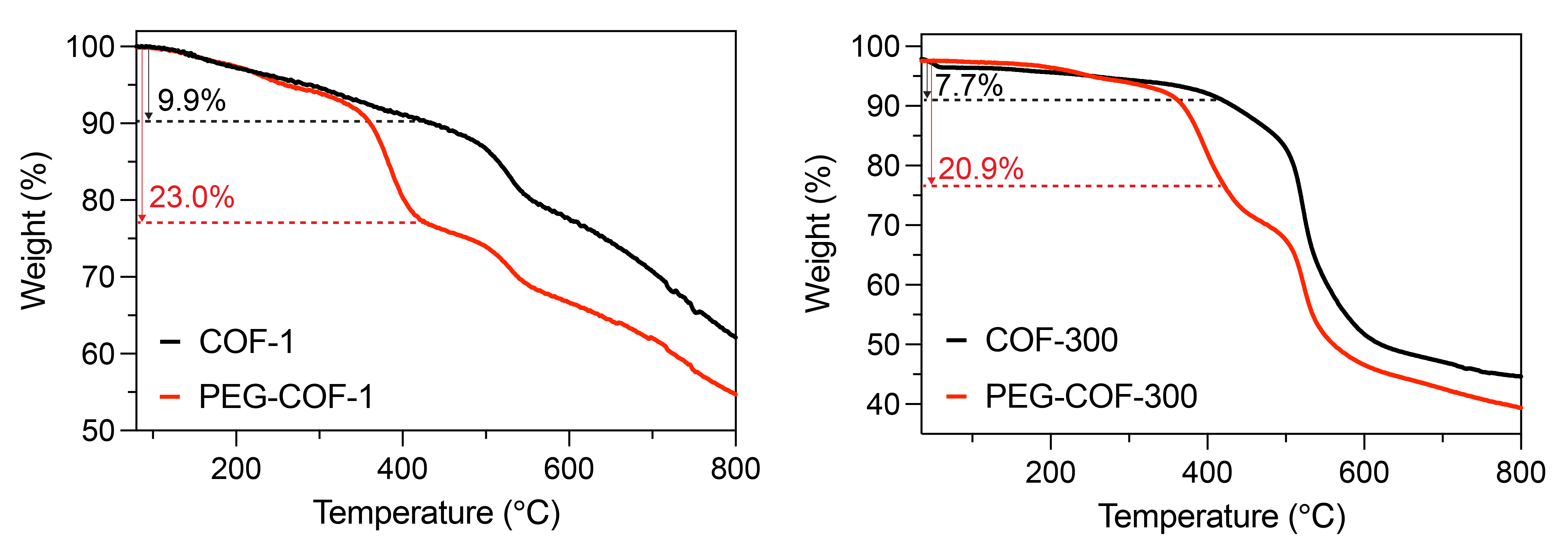


**Figure S11.** Thermogravimetric analysis (TGA) curves of nCOFs and PEG-modified nCOFs.


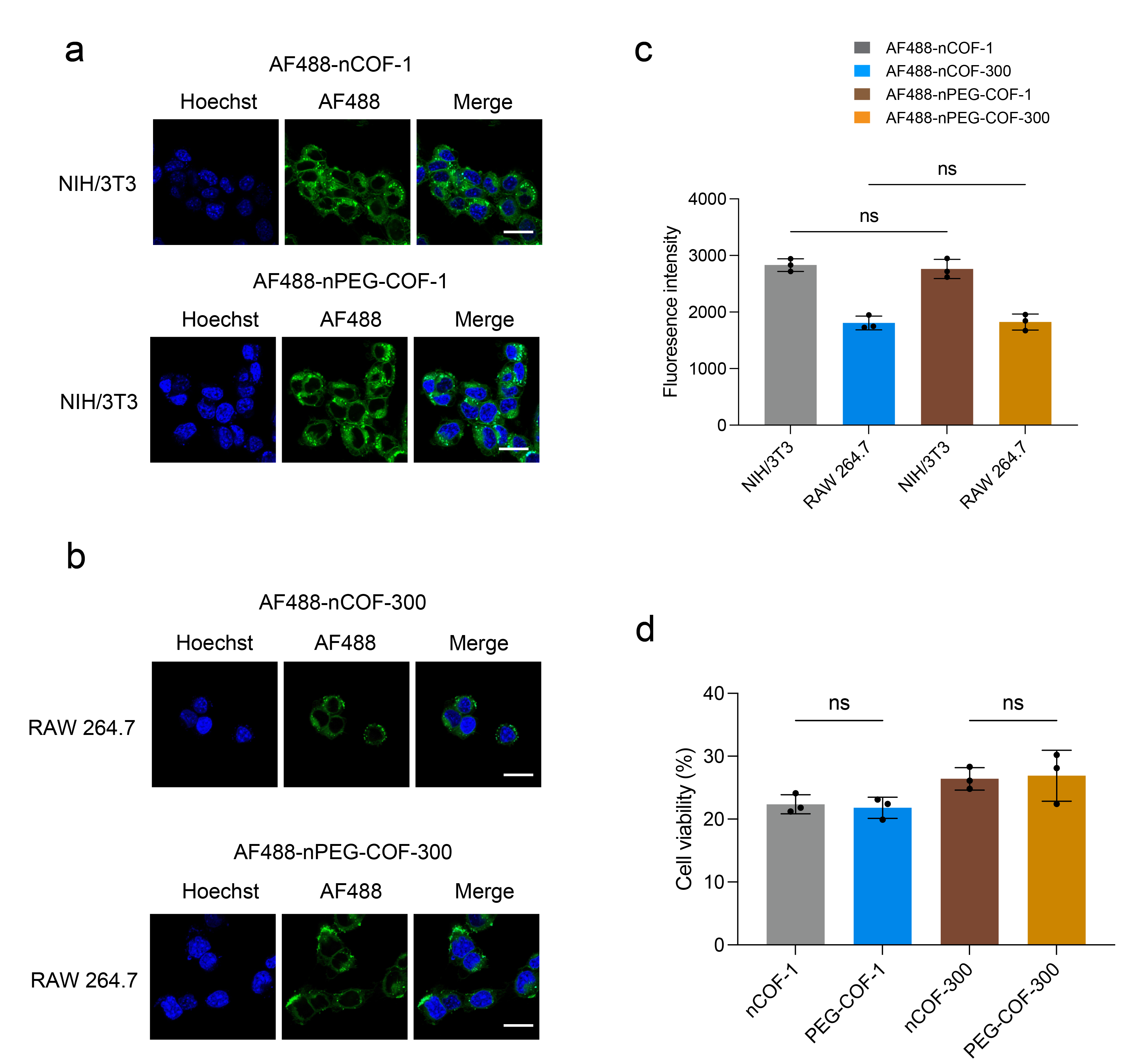


**Figure S12**. a) Confocal laser scanning microscopy (CLSM) images for NIH/3T3 cells or b) RAW 264.7 cells after incubating with AF488-nCOF-1 or AF488-nPEG-COF-1 for 2 h, respectively (n = 3, Scale bar = 10 μm). c) Flow cytometry quantify results for RAW 264.7, or NIH/3T3 cells after incubating with nCOFs samples (n = 3, data are presented as mean ± s.e.m.). d) Inhibitory effects of nCOFs samples (200 μg mL-1) on the proliferation of MG-63 cells for 72 h incubation as evaluated by MTT assay (n = 3, data are presented as mean ± s.e.m.).


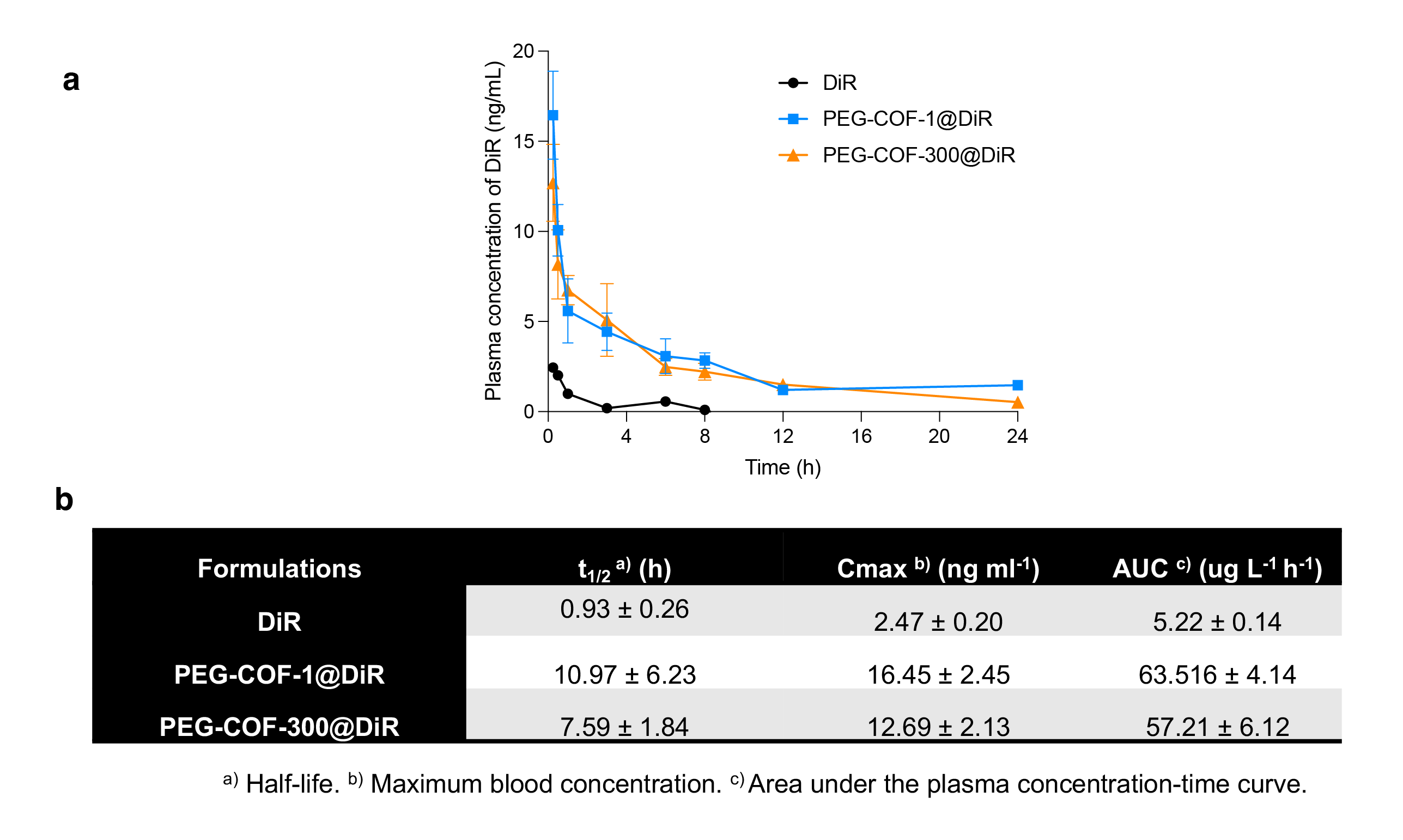


**Figure S13.** a) In vivo pharmacokinetics curves and b) parameters of DiR, PEG-COF-1@DiR, and PEG-COF-300@DiR (IV injection) (n = 5, data are presented as mean ± s.e.m.).


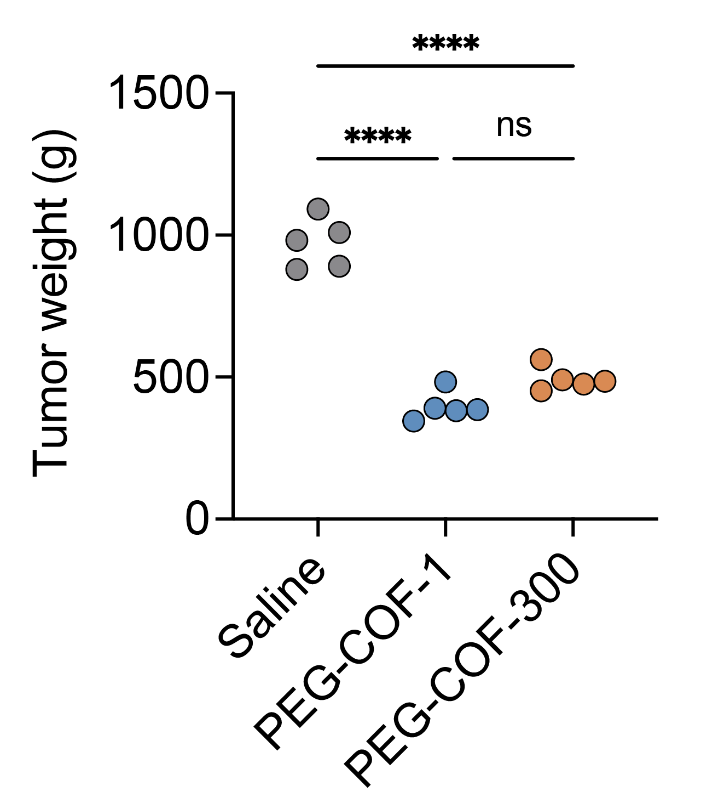


**Figure S14.** Tumor weight of MG-63 tumor-bearing mice model after various treatments at day 14 (n = 5). Statistical significance was calculated via one-way ANOVA with a Tukey post-hoc test. **** p< 0.0001 versus control.


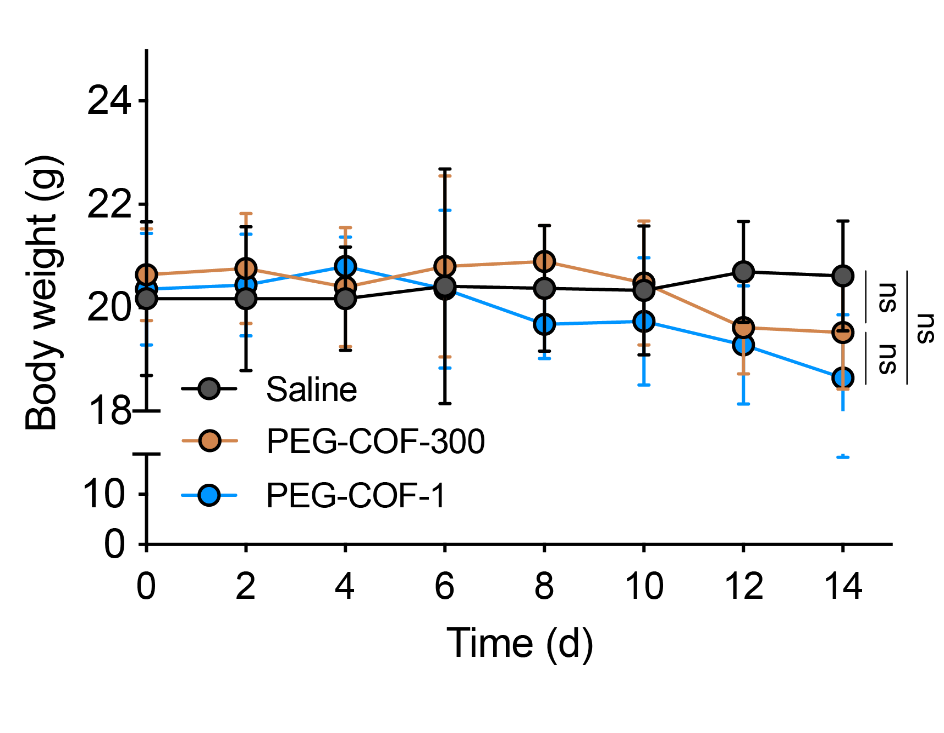


**Figure S15.** Body weight of MG-63 tumor-bearing mice model after various treatments (n = 5, data are presented as mean ± s.e.m.). Statistical significance was calculated via one-way ANOVA with Dunnett’s multiple comparison test.


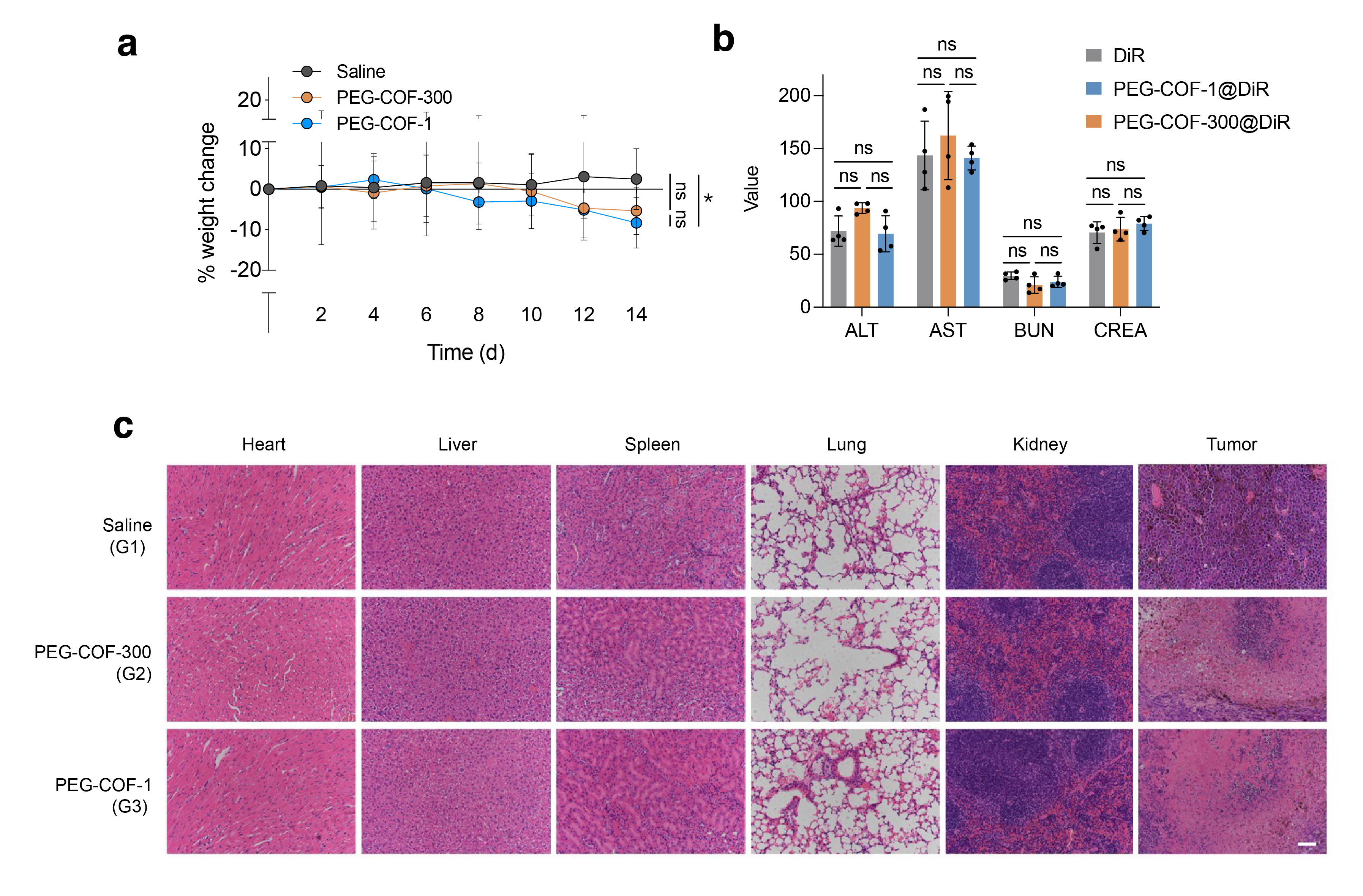


**Figure S16.** a) Body weight changes after various treatments (n = 5). b) Hepatorenal function parameters of blood obtained at day 14 (n = 4). c) H&E staining for major organs slices at day 14 (n = 3, Scale bar = 1 mm). Statistical significance was calculated via one-way ANOVA with a Tukey post-hoc test (Figure S16b) or with Dunnett’s multiple comparison test (Figure S16a).* p < 0.05, ** p < 0.01, ***p< 0.001, **** p< 0.0001 versus control.

**Figure S17.** a) Size and b) size distribution of COF-1 and COF-300 after 24-hour incubation in pH 4.5 acetic acid at 37°C. Particle sizes and size distributions were measured by dynamic light scattering (DLS) using a Malvern Zetasizer.

**Reference:**

1. Ye, H. et al. Bioinspired nanoplatelets for chemo-photothermal therapy of breast cancer metastasis inhibition. *Biomaterials* **206**, 1-12 (2019).
